# Sequentially targeting and intervening mutual Polo-like Kinase 1 on CAFs and tumor cells by dual targeting nano-platform for cholangiocarcinoma treatment

**DOI:** 10.7150/thno.70557

**Published:** 2022-05-09

**Authors:** Yue Zhou, Lei Xu, Zhangding Wang, Hongwen Liu, Xiang Zhang, Chuanjun Shu, Meng Zhang, Ting Wang, Xinyun Xu, Xiaohong Pu, Jian He, Pin Wang, Yudong Qiu, Guifang Xu, Xiaoping Zou, Yun Zhu, Lei Wang

**Affiliations:** 1 Department of Gastroenterology, Nanjing Drum Tower Hospital, The Affiliated Hospital of Nanjing University Medical School, Nanjing 210008, Jiangsu Province, China.; 2 Department of Gastroenterology, Nanjing Drum Tower Hospital, Drum Tower Clinical Medical College of Nanjing Medical University, Nanjing 210008, Jiangsu Province, China.; 3 Department of Pharmacy, Nanjing Drum Tower Hospital, Drum Tower Clinical Medical College of Nanjing Medical University, Nanjing 210008, Jiangsu Province, China.; 4 Department of Bioinformatics, School of Biomedical Engineering and Informatics, Nanjing Medical University, Nanjing 211166, Jiangsu Province, China.; 5 Department of Pathology, Nanjing Drum Tower Hospital, The Affiliated Hospital of Nanjing University Medical School, Nanjing 210008, Jiangsu Province, China.; 6 Department of Nuclear Medicine, Nanjing Drum Tower Hospital, The Affiliated Hospital of Nanjing University Medical School, Nanjing 210008, Jiangsu Province, China.; 7 Department of Hepatopancreatobiliary Surgery, Nanjing Drum Tower Hospital, The Affiliated Hospital of Nanjing University Medical School, Nanjing 210008, Jiangsu Province, China.; 8 Nanjing Medical Center for Clinical Pharmacy, Nanjing 210008, Jiangsu Province, China.

**Keywords:** polo-like kinase 1, cholangiocarcinoma, stroma, drug delivery system, targeting, CAFs

## Abstract

**Rationale**: Synergistic treatment strategies for two or more drugs have gradually developed as the main options in clinics for cholangiocarcinoma (CCA) owing to the complicated crosstalk between the tumor and stroma. However, the different synergetic mechanisms pose great challenges to the dosages and order of administration of drugs. Thus, a strategy for exploring and intervening in mutual targets derived from stromal cells and cholangiocarcinoma cells was proposed.

**Methods**: Genes with overexpression patterns in tumors and displaying a significant association with overall survival were identified from RNA-seq data of human CCA patients and CCA mouse models. Western blotting, qRT-PCR, immunofluorescence (IF), colony formation and flow cytometry assays were conducted to determine the biological roles of the key oncogene in cholangiocarcinoma and stromal cells respectively. Additionally, a dual-targeting drug delivery system (AA-HA-ODA) for cancer-associated fibroblasts (CAFs) and tumor cells was constructed to verify the effectiveness of intervening the screened genes *in vivo*.

**Results**: Polo-like kinase 1 (PLK1) was verified to play vital role in the malignant proliferation of CCA by regulating the cell cycle pathway. PLK1 also decreased stromal production by regulating the CAF phenotype. In addition, a PLK1 inhibitor (Ro3280) loaded dual-targeting drug delivery system (AA-HA-ODA) was prepared and exhibited high affinity for CAFs and cholangiocarcinoma cells. The *in vivo* distribution pattern and antitumor efficacy of AA-HA-ODA/Ro also verify the effectiveness of inhibiting PLK1 in CCA *in vivo*.

**Conclusion**: In summary, PLK1 is a mutual target derived from tumor cells and stroma due to its crucial role in the proliferation of tumor cells and stroma regulation in CAFs, which might provide enlightenment for multitarget treatment strategies and guidance for clinical cholangiocarcinoma treatment.

## Introduction

Cholangiocarcinoma (CCA), the second most common primary hepatic malignancy after hepatocellular carcinoma, is often diagnosed when the disease is already in advanced stages, which highly compromises therapeutic options, resulting in a dismal prognosis^1^, ^2^. Regarding the treatment of CCA, the main studies normally focused on single potential genes or signaling pathways acting on tumor cells, which indeed achieved great progress. However, with the insight of the tumor microenvironment for tumorigenesis, the interaction between stroma and tumor cells was recognized as the pivotal contributor to the insufficient antitumor therapy, high recurrence and metastasis^3^, ^4^.

CCA is characterized by reactive desmoplastic stroma that crosstalk with cholangiocarcinoma cells and the secreting factors. Cancer-associated fibroblasts (CAFs), the main resource of the tumor stroma, are normally recruited and activated persistently by cholangiocarcinoma cells, in response to the activation of stromal cell derived factor 1 (SDF1), transforming growth factor β (TGF-β) and extracellular matrix (ECM) proteins^5^-^7^. In turn, CAFs can promote the proliferation and invasive ability of cholangiocarcinoma cells directly^3^, ^8^-^10^. Regarding the prominent tumor promoting effect of stroma, the treatment of single-target exploitation for cholangiocarcinoma cells is normally characterized by low efficacy and high recurrence. Thus, synergistic treatment strategies for two or more drugs have gradually been developed as the main clinical options for cholangiocarcinoma^11^-^15^. However, the dosages and order of administration pose a huge challenge because of the complicated synergistic mechanism of combination drugs and heterogeneous pathologic structures in CCA. Hence, the exploration of mutual targets derived from cholangiocarcinoma cells and stroma may hold great potential for CCA treatment.

In this study, genomic and transcriptomic studies revealed that cell cycle-related genes were significantly enriched in CCA 16, 17. Specifically, overexpression of PLK1 was detected in both resection specimens of CCA patients and mouse models and was highly correlated with poor prognosis. In addition, PLK1 has also been found to regulate hepatic stellate cell activation to form CAFs phenotype and promote liver fibrosis^18^. The TGF-β signaling pathway is mainly involved in the activation of fibroblasts during fibrogenesis, and activated PLK1, as a promoter of fibroblast differentiation, could form a positive feedback loop with TGF-β 19-22, jointly illuminating that PLK1 plays vital roles in the regulation of both CCA tumor cells and CAF-derived stroma, making it an ideal target for cancer-specific silencing. Thus, taking advantage of targeting and intervening in mutual PLK1 on cholangiocarcinoma cells and CAFs and exploring the underlying mechanism hold great potential for treating CCA.

Notably, the therapeutic dilemmas of complicated crosstalk between tumor cells and stroma in desmoplastic tumors are often accompanied by pathological stroma barrier, that tumor vessels are normally embedded into the rich and dense stroma in desmoplastic tumors including CCA, which reduces accessibility of intratumorally delivered drugs 23. Briefly, the resulting off-targeting effect of delivered drugs by the pathological stroma barrier could further compromise the effectiveness of the proposed antitumor strategy including the only tumor cells targeting drugs 24-26. Thus, ideal drug delivery system (DDS) that exhibit affinity for CAFs and tumor cells might hold potential for sequentially delivering PLK1 inhibitor (Ro3280). The sigma receptor, overexpressed in many malignant tumors and CAFs, has high affinity with anisamide derived ligands 27-29. Biocompatible, biodegradable, non-toxic, and non-immunogenic hyaluronic acid (HA) and octadecylamine are widely used in anticancer drug delivery respectively 30, 31. Thus, a targeted drug delivery system (AA-HA-ODA) taking hyaluronic acid as the basic skeleton and PEG linked sigma receptor ligand anisamide (AA) modified on the surface of micelles was constructed, which could sequentially target CAFs and obtain access to intratumoral CCA tumor cells 28, 30, 32, 33. Next, a PLK1 inhibitor (Ro3280) was encapsulated to prepare the AA-HA-ODA/Ro and evaluate the effectiveness of antitumor and stromal regulation in organoids, multicellular spheroids *in vitro* and AKT/YapS127A plasmid-induced primary CCA mouse models *in vivo*. The sequential targeting capacity of AA-HA-ODA was also verified. In summary, the feasibility of the proposed strategy that targeting and intervening the mutual PLK1 on both CAFs and the inner CCA tumor cells by targeting AA-HA-ODA sequentially was verified, which might provide enlightenment for clinical cholangiocarcinoma treatment.

## Materials and Methods

### Materials

Hyaluronic acid (HA), with a molecular weight (MW) of 3000-1000Da, was purchased from the Shandong Freda Biotechnology Co., Ltd. (Shandong, China). 1-Hydroxy-5-pyrrolidinedione (NHS), 1-ethyl-3-(3-dimethylaminopropyl) carbodiimide (EDC), 4-methoxybenzoic acid, octadecylamine, indocyanine green (ICG), Dimethyl sulfoxide (DMSO) were all purchased from Aladdin Reagent Database Inc (Shanghai, China). Polyethylene glycol (PEG) with 2000 molecular weight was purchased from Shanghai Yare Biotechnology Co., Ltd (Shanghai, China). 1,1'-Dioctadecyl-3,3,3',3'-tetramethylindocarbocyanine perchlorate (DiI) was purchased from Beyotime Institute of Biotechnology (Jiangsu, China). All inhibitors used were purchased from MedChemExpress (Monmouth Junction, NJ, USA).

### Cell lines and cell culture

HuCCT1 cells were purchased from JCRB, Osaka, Japan. HCCC98010, RBE, NIH/3T3 mouse embryonic fibroblast cells and LX2 hepatic stellate cells were purchased from the Institute of Biochemistry and Cell Biology, Shanghai Institutes for Biological Sciences, Chinese Academy of Sciences, Shanghai, China. NIH/3T3 cells were cultured in DMEM (Biological Industries, Cromwell, CT, United States) and the other cells were cultured in RPMI-1640 (Invitrogen, Waltham, MA, USA) medium. All cells were maintained at 37 °C with 5% CO_2_ and cultured with 10-20% fetal bovine serum (Biological Industries, Cromwell, CT, USA), penicillin (Invitrogen) (100 U/mL), and streptomycin (Invitrogen) (100 U/mL).

### Preparation of human cholangiocarcinoma specimens and mouse model

Thirty cases of pathologically confirmed CCA tissues and the corresponding noncancerous tissues were collected from patients with radical surgery at the Nanjing Drum Tower Hospital, the Affiliated Hospital of Nanjing University Medical School (Nanjing, Jiangsu, China). All experiments involving human specimens were approved by the Ethical Committee of Medical Research, Nanjing Drum Tower Hospital, Affiliated Hospital of Nanjing University Medical School.

Female FVB/N mice (5 to 6 weeks old) were purchased from Beijing Vital River Laboratory Animal Technology and housed in in a specific pathogen-free (SPF) environment. To generate the CCA model, we injected 20μg pT3-EF1α-HA-myr-AKT and 30μg pT3-EF1α-YapS127A plasmids in FVB/N mice via hydrodynamic injection. All plasmids including Sleeping beauty transposase (SB), pT3-EF1α-HA-myr-AKT and pT3-EF1α-YapS127A were kind gifts from Dr. Zhang (Department of Pathology, Eastern Hepatobiliary Surgery Hospital, Second Military Medical University, Shanghai, China) 34-37. Then, tumor tissues were collected, and stained with hematoxylin and eosin (HE). All experiments utilizing animals were reviewed and approved by the Ethical Committee and Animal Welfare Committee of Drum Tower Hospital, the Affiliated Hospital of Nanjing University Medical School.

### Immunohistochemistry and Immunofluorescence

Tumor tissues were embedded in paraffin. Except for target detection, the IHC followed standard procedures. Slides of the tumors were deparaffinized, and blocked, and the primary antibody was incubated overnight at 4 °C. Finally, the secondary antibody labeled with biotin for immunohistochemistry and labeled with fluorescent for immunofluorescence were incubated for 1 hour at room temperature.

### Bioinformatic analysis

The “limma” package in the statistical software R was used to screen the differentially expressed genes (DEGs) between cancerous and noncancerous samples, with the cut‐off criteria of adjusted P < 0.05 and |log_2_FC| > 1. The Database for Annotation Visualization and Integrated Discovery (DAVID) online tool (https://david.ncifcrf.gov/) was used to perform functional and pathway enrichment analyses. We used the STRING (http://string‐db.org) online database to obtain a protein-protein interaction (PPI) network, which was visualized by Cytoscape software (version 3.3.0). Node degree values were further calculated by Cytoscape software. Univariate Cox analysis of overall survival (OS) was performed to calculate the relationship of genes with overall survival (OS). Genes with prognostic significance (p < 0.05) were further selected and validated through LASSO regression using the R project “glmnet” package, and the Kaplan-Meier method and log-rank test were used to detect potential prognostic factors. Four cholangiocarcinoma transcriptome gene expression profile datasets were downloaded from the GEO database (https://www.ncbi.nlm.nih.gov/geo/): GSE119336, GSE32879, GSE107943, and GSE76297. Transcriptome data and clinical information from the TCGA database were downloaded via the UCSC Genome Browser (https://genome.ucsc.edu).

### Molecular Docking Method

The three-dimensional structures of the proteins in this study was predicted by AlphaFold (https://alphafold.com/). Then, the largest possible binding pocket of proteins was then predicted by Discovery Studio 3.0, respectively. These predicted pockets were utilized to construct an initial coarse model of the protein-molecules complex. Then, interaction between small molecules and protein were by further explored by Discovery Studio 3.0. High-quality 3-D images of structures were drawn by Discovery Studio 3.0^38^-^40^. Based on binding energy scores, the complex with the lowest score was chosen as the finally structure.

### Colony formation assay

CCA cells were counted and seeded in six-well plates at the concentration of 500 cells/well in complete medium containing 10% fetal bovine serum, and treated with different reagents for indicated time. After 14 days, the cells were fixed and stained. Finally, colonies with more than 50 cells were counted and representative images were captured. Three independent colony formation assays were performed.

### Apoptosis assay and cell cycle analysis via flow cytometry

After treatment for the indicated times, samples were detected by flow cytometry. Apoptosis was detected with an Annexin V-FITC Apoptosis Detection Kit, and the cell cycle was detected with a BD Cycletest Plus DNA Reagent Kit, following the manufacturer's instructions. Finally, the data were analyzed with FlowJo software. Samples were measured by a BD FACS Canto II flow cytometer (BD Biosciences, CA).

### Western blot

Samples, including tissues and cells, were lysed in RIPA lysis buffer (Biosharp) mixed with protease and phosphatase inhibitor cocktail (Roche Diagnostics GmbH, Mannheim, Germany) and phenylmethylsulfonyl fluoride (PMSF) (Biosharp, Hefei, China) for 15 min on ice. Then, the proteins were subjected to western blotting according to standard protocols. The antibodies used were as follows: PLK1 (CST, 4513s), α-SMA (Abcam, ab7817), FOXM1 (Santa Cruz, sc-376471), YAP (CST, 14074T), AKT (Abcam, ab8805), cleaved caspase-3 (CST, 9664s), cleaved PARP (CST, 5625s), C-MYC (Abcam, ab32072) and β-actin (Sigma, A5441).

### Synthesis and characterization of the AA-HA-ODA

**EDC (**0.550 g), NHS (0.330 g) and HA (0.240 g) were dissolved in 30 mL of mixed solvent (DMSO:H_2_O = 4:1) and magnetically stirred at 60 ℃ under condensation reflux for 1 hour. Then, 0.04 g octadecylamine was dissolved in 4 ml of anhydrous ethanol, added to the above reaction solution, and magnetically stirred at 60 ℃ under condensation reflux for 24 h. The final reaction solution was put into a dialysis bag for dialysis against deionized water overnight and lyophilized. The lyophilized product was suspended in ethanol and centrifuged at 6000 rpm for 10 min, and the supernatant was discarded to remove the octadecylamine. After three repetitions, the precipitate was re-dissolved in deionized water to obtain the final product HA-ODA polymer by lyophilization.

To acquire the AA-HA-ODA polymer, 0.0152 g of 4-methoxybenzoic acid, 0.2 g PEG, 0.057 g EDC and 0.034 g NHS were weighed, dissolved in 4 mL dichloromethane, and reacted overnight at 40 °C under condensation reflux. Then, the intermediate was obtained by spin evaporation. Next, 0.180 g EDC, 0.110 g NHS and 0.080 g HA-ODA were dissolved in 20 mL mixed solvent (ethanol:H_2_O = 1:1) and activated for 1 h under magnetic stirring at 60 ℃. The above intermediates dissolved in 1 mL ethanol were subsequently added, and the reaction was continued at 60 ℃ for 24 h. The synthesized products were dialyzed and purified to obtain AA-HA-ODA. 1H-NMR was used to verify the polymer structure. Pyrene fluorescence was used to measure the critical micelle concentration (CMC) of HA-ODA and AA-HA-ODA 41.

### Preparation of AA-HA-ODA/Ro

HA-ODA (4 mg) and AA-HA-ODA (4 mg) were dissolved in 2 mL deionized water and ultrasonicated for 5 min (30 W, working for 3 s, stopping for 3 s). According to the dosage of 10% (w/w), an appropriate volume of Ro3280/DMSO solution was added to the micelle solution and stirred at room temperature for 20 min. The solution was transferred to a dialysis bag for dialysis in deionized water for 24 h. Finally, the residual Ro3280 was removed by centrifugation. The drug content was determined by UV-Vis spectrophotometry. The dialysis bag method was used to evaluate drug release profile of HA-ODA/Ro and AA-HA-ODA/Ro *in vitro* 11.

### Cellular uptake and penetration evaluation *in vitro*

Cells were inoculated in 24-well plates with preplaced slides overnight at 37 °C, and then HA-ODA/DiI and AA-HA-ODA/DiI were added for further incubation. The loading method of DiI was performed as described by Ro3280. After incubation for 12 h, the nuclei were stained with DAPI for 10 min, washed three times with PBS and fixed at room temperature with 4% paraformaldehyde. The fluorescence intensity was observed by confocal microscopy.

The agarose solution (1.5%, w/v) dissolved in deionized water (50 μl) was seeded into a 96-well plate. After cooling at 37 °C for one hour, 190 μL fresh cell culture medium was added to each well, and 10 μL suspension droplets containing 1000 HuCCT1 cells and NIH/3T3 cells were added to the 96-well culture plate 42, 43. The cells slowly aggregated and grew into a 3D multicellular tumor sphere (MCTSs). When the diameter of the MCTSs reached a value suitable for the experiment, 10 μL free DII, HA-ODA/DiI and AA-HA-ODA/DiI with the same concentration were added to the culture medium of the MCTSs. After two days of incubation, 10 μl DAPI was added to each well to stain the nucleus for 10 min. Then, MCTSs were collected, washed with PBS 3 times and fixed with 4% paraformaldehyde. Finally, the fluorescence intensity of the MCTSs was observed by confocal microscopy.

For antitumor therapy *in vitro*, Ro3280, HA-ODA/Ro3280 and AA-HA-ODA/Ro3280 were diluted to 15 μM with fresh medium. When MCTSs met the experimental requirements, they were divided into 4 groups. After administration, the MCTSs were observed and photographed with a white light microscope every day. When MCTSs showed obvious blurred edges and blackened centers, the cell pellets were collected and digested into a single-cell suspension for apoptosis detection.

### Biodistribution of AA-HA-ODA *in vivo*

The CCA murine models were established according to the methods described. To observe the distribution of AA-HA-ODA *in vivo*, ICG was used to label HA-ODA and AA-HA-ODA. Free ICG, HA-ODA/ICG and AA-HA-ODA/ICG (120 μL) were injected intravenously. Mice were imaged at the indicated times using an *in vivo* imaging system (IVIS Lumina XR, Caliper, USA). The distribution of nanoparticles was evaluated according to ICG fluorescence intensity.

To assess the targeting effect of nanoparticles on tumor tissues, red fluorescent free DII and DII-labeled nanoparticles were injected intravenously. Seventy-two hours after injection, livers were excised and fixed with 4% paraformaldehyde. Subsequently, the liver tissues were stained with CK19 (labeled tumor cells) and α-SMA (labeled fibroblast cells) by immunofluorescence staining. The nuclei were stained with DAPI. Finally, the fluorescence of tissue sections was observed and imaged by confocal microscopy.

### *In vivo* antitumor efficacy

The CCA models mentioned were used to evaluate the antitumor efficacy of AA-HA-ODA/Ro. First, CCA models were randomly divided into four groups: Control, Ro3280, HA-ODA/Ro3280 and AA-HA-ODA/Ro3280. After 6 days of administration, body weight and antitumor efficacy were monitored. On Day 21, the mice were sacrificed. The resected tumor masses were fixed in 4% paraformaldehyde solution for HE, Masson and IHC staining analysis.

### Statistical Analysis

The results are presented as the mean ± standard derivation. The analysis of difference was examined by two-tailed Student's t test. A p value of p < 0.05 was considered statistically significant.

## Results and discussion

### Cholangiocarcinoma is a desmoplastic tumor that tumor cells are trapped by compact stroma

**Figure 1:**
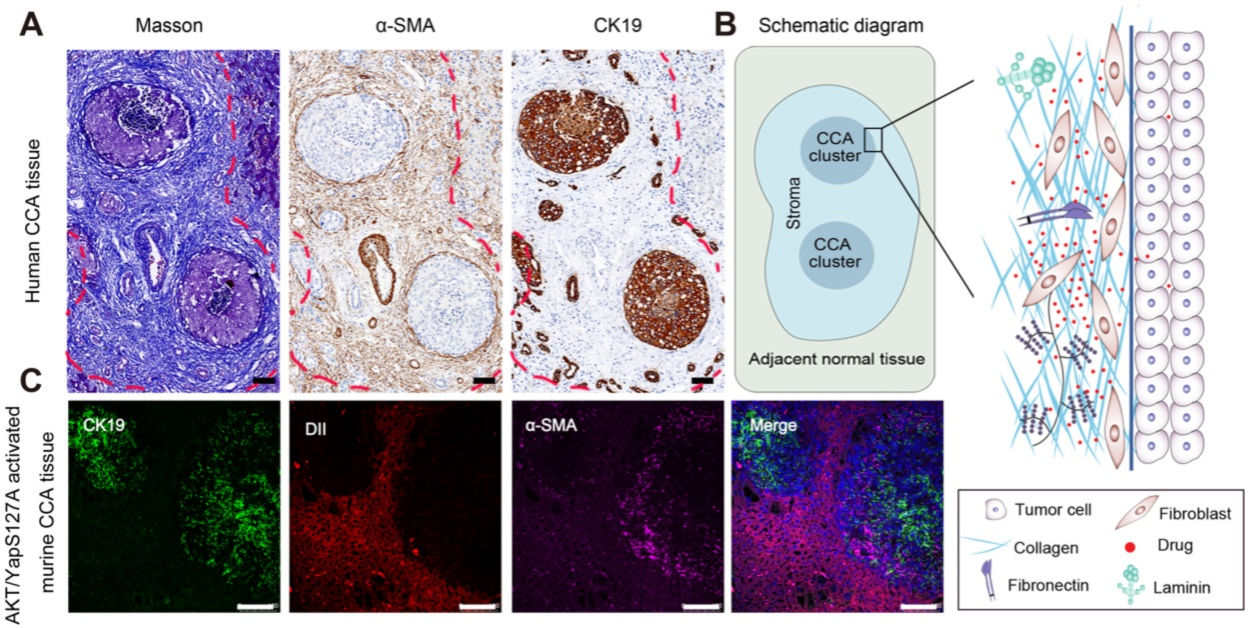
** Pathological stromal barrier and the impeding drugs distribution in CCA tissue.** A) Desmoplastic stroma in human CCA by masson and IHC staining for α-sma and CK19. B) Schematic diagram for the stroma barrier and the detained stromal components distribution pattern in human CCA. C) Evaluation of stroma feature and resulting drugs distribution pattern in AKT/YapS127A induced murine CCA model, scale bar: 100μm.

With the development of "seed" and "soil" theory, the tumor microenvironment is no longer regarded as s bystander. Instead, great importance has been recognized by researchers 44, 45. For instance, the stroma comprises of the major noncancerous components, which interact with tumor cells to induce drug resistance, metastasis and recurrence 13, 23. In this study, CCA tissues from patients were collected and evaluated by Masson staining, IHC staining of CK19-labeled tumor cells and α-SMA- labeled fibroblasts as shown in Figure 1A. The stromal components around tumor clusters in CCA are abnormally rich, showing a protecting "wall" for exogenous distributed substances. Notably, when the tumor clusters become larger or close to each other, the surrounding stroma at different positions will be linked and form a larger barrier "wall" structure (shown by the red dotted lines), further hindering the distribution and penetration of the administered drug. A schematic diagram of the stromal barrier and the distribution pattern of the retained stromal components in CCA are shown in Figure 1B.

To further investigate the histological structure of CCA, we constructed an AKT/YapS127A CCA mouse model by hydrodynamic injection transfection. Subsequent H&E staining, accompanied by immunochemical analysis of CK19 and α-SMA confirmed the formation of cholangiocarcinoma lesions (Figure S1A-C). Moreover, immunofluorescence analysis revealed that the green fluorescently labeled CCA tumor clusters were tightly surrounded by purple fluorescence labelled CAFs (Figure S1C and Figure 1C). To evaluate the drug penetration pattern *in vivo*, a red fluorescence probe (DiI) was utilized to visualize the distribution pattern in CCA by tail intravenous injection. The pathological barrier severely hindered the distribution of red fluorescent probes in the tumor (the red fluorescent intensity in the tumor tissue area was significantly lower than that in the surrounding normal liver tissue), confirming the tumor-promoting and treatment-resistant effects of CCA (Figure 1C).

### PLK1 is pivotal in cholangiocarcinoma tumorigenesis and associated with poor prognosis in CCA patients

**Figure 2:**
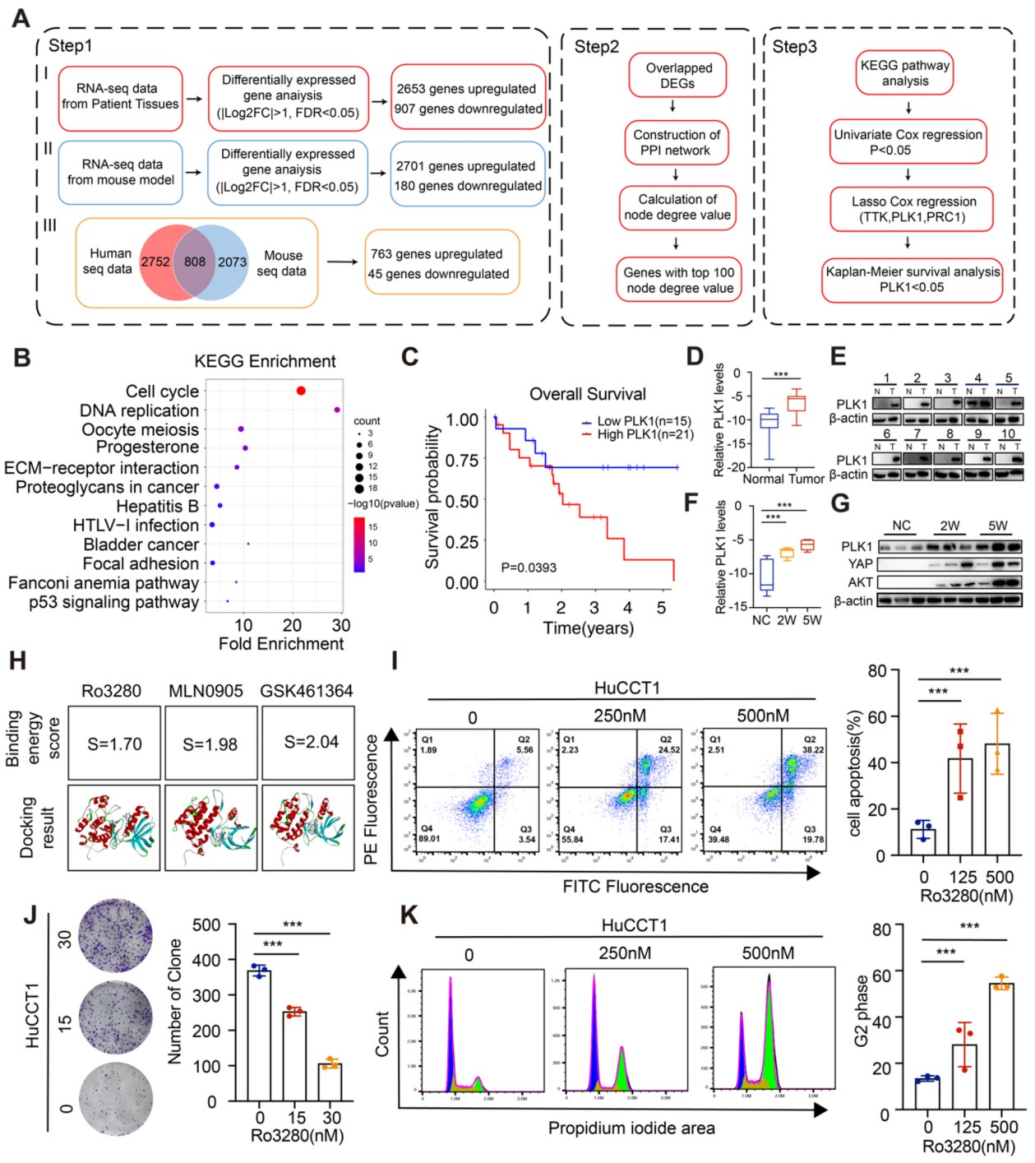
** Pivotal role of PLK1 in cholangiocarcinoma tumorigenesis and the association with poor prognosis in CCA patients.** A) Flow chart of the study design. B) KEGG enrichment analysis for the genes with top 100 node degree value in the PPI network. C) Kaplan-Meier survival curve in the high and low PLK1 expression groups. D-E) .PLK1 mRNA (Relative levels normalized to GAPDH (log2)) and protein levels in CCA cancerous specimen and paired noncancerous specimen by qRT-PCR, n = 10. F-G) PLK1 mRNA (Relative levels normalized to GAPDH (log2)) and protein levels in specimen from CCA murine models at different stages by qRT-PCR (2 weeks & 5 weeks post injection). H) Binding energy score of Ro3280, MLN0905 and GSK461364 with PLK1 structure, respectively. I) Cell apoptosis assays performed by flow cytometry after treatment of Ro3280 (left panel), quantification of the cell apoptosis assay results (right panel). J) Representative images of colony formation assay in HuCCT1 after treatment of Ro3280 (left panel), quantification of the colony formation assay results (right panel). K) Cell cycle assays performed by flow cytometry after treatment of Ro3280 (left panel), quantification of the cell cycle assay results (right panel).

To investigate critical biological processes in CCA carcinogenesis, by conjointly analyzing the differentially expressed transcripts from RNA-seq data of CCA mouse models and human CCA patients, 808 overlapping differentially expressed genes (DEGs) were screened out (Figure 2A and Figure S2). Then, the protein interaction (PPI) network was constructed by the STRING online database as shown in the detailed flow chart in Figure 2A. Of note, Kyoto Encyclopedia of Genes and Genomes (KEGG) pathway analyses of genes with the top 100 node degree values clustered the majority of genes in the Cell cycle (Figure 2B and Figure S3). We then performed univariate Cox regression analysis and the LASSO algorithm combined with the prognostic information in the TCGA dataset to determine the effects of these 100 transcripts on clinical prognosis, and three genes (PLK1, TTK, PRC1) were identified (Figure S4A-E). Specifically, only the CCA patients with high levels of PLK1 had worse OS according to the Kaplan-Meier survival analysis (Figure 2C and Figure S4F-G), indicating the pivotal role of PLK1 in CCA.

Then, the PLK1 expression in the TCGA dataset and four additional CCA disease datasets (GSE107943, GSE119336, GSE32879 and GSE76297) was detected, and the results showed obviously increased PLK1 expression in CCA cancerous tissues compared with normal tissues (Figure S5A). Subsequently, we performed immunofluorescence staining in tumor tissues and normal liver tissues. Notably, overexpressed PLK1 was observed in the tumor sites (Figure S5B). High levels of PLK1 were also confirmed in 10 human CCA tissues compared to the corresponding normal tissues by qRT-PCR and western blotting assays (Figure 2D-E). Furthermore, we assessed PLK1 expression levels in different stages in the CCA model. The results showed that the expression level of PLK1 increased gradually with tumor progression (Figure 2F-G). Taken together, these results suggest that the expression of PLK1 is upregulated in CCA tissues and could act as an independent prognostic factor for CCA.

To evaluate the biological function of PLK1 in CCA, PLK1 inhibitors were screened and three drugs, MLN0905, Ro3280 and GSK461364, were adopted according to the cell viability after drug treatment. The results in Figure S6A showed significantly inhibitory effect on CCA cell lines. Molecular docking method was applied to further screen the candidate PLK1 inhibitor, in which the binding energy score calculated by computer simulation reflect the binding stability between PLK1 inhibitors and PLK1 as reported in our previous works 38-40. In specific, Ro3280 with the lowest score was chosen for the following assay (Figure 2H and Figure S6B-D). In addition, both the colony-forming capacity and Annexin-V/FITC assay on HuCCT1 and RBE cells exhibited dose-dependent pharmacological effect (Figure 2I-J, Figure S7A-B). Moreover, the protein levels of the cleaved form of poly (ADP-ribose) polymerase 1 (PARP1) and Caspase3, biomarkers for apoptosis, were detected and showed an increasing trend in HuCCT1 and RBE cells after treatment with Ro3280 (Figure S7C). To further examine the roles of PLK1 in the cell cycle, a flow cytometry assay was performed. The percentage of CCA cells arrested in the G2-M phase increased sharply with Ro3280 treatment (Figure 2K and Figure S7D). Intriguingly, the protein levels of several cell cycle-related genes, including FOXM1, BUB1, and CMYC, were correspondingly decreased upon pharmacological inhibition of PLK1 by Ro3280 (Figure S7E). Collectively, these results indicated the critical pharmacological effect of the PLK1 inhibitor on cholangiocarcinoma cells.

### PLK1 is strongly associated with activation of fibroblasts and stromal deposition

In research studies, PLK1 has been found to be involved in regulating myofibroblast and hepatic stellate cell activation and liver fibrosis^18^, ^19^. In addition, activated PLK1 could form a positive feedback loop with TGF-β which is mainly involved in the activation of fibroblasts during fibrogenesis, illuminating that PLK1 might be a potential target for regulating the stroma^19^-^21^. To verify whether PLK1 could satisfy the demand for the proposed strategy that targets mutual targets derived from tumor cells and stroma, the regulatory role of PLK1 for stromal cells was further explored.

**Figure 3:**
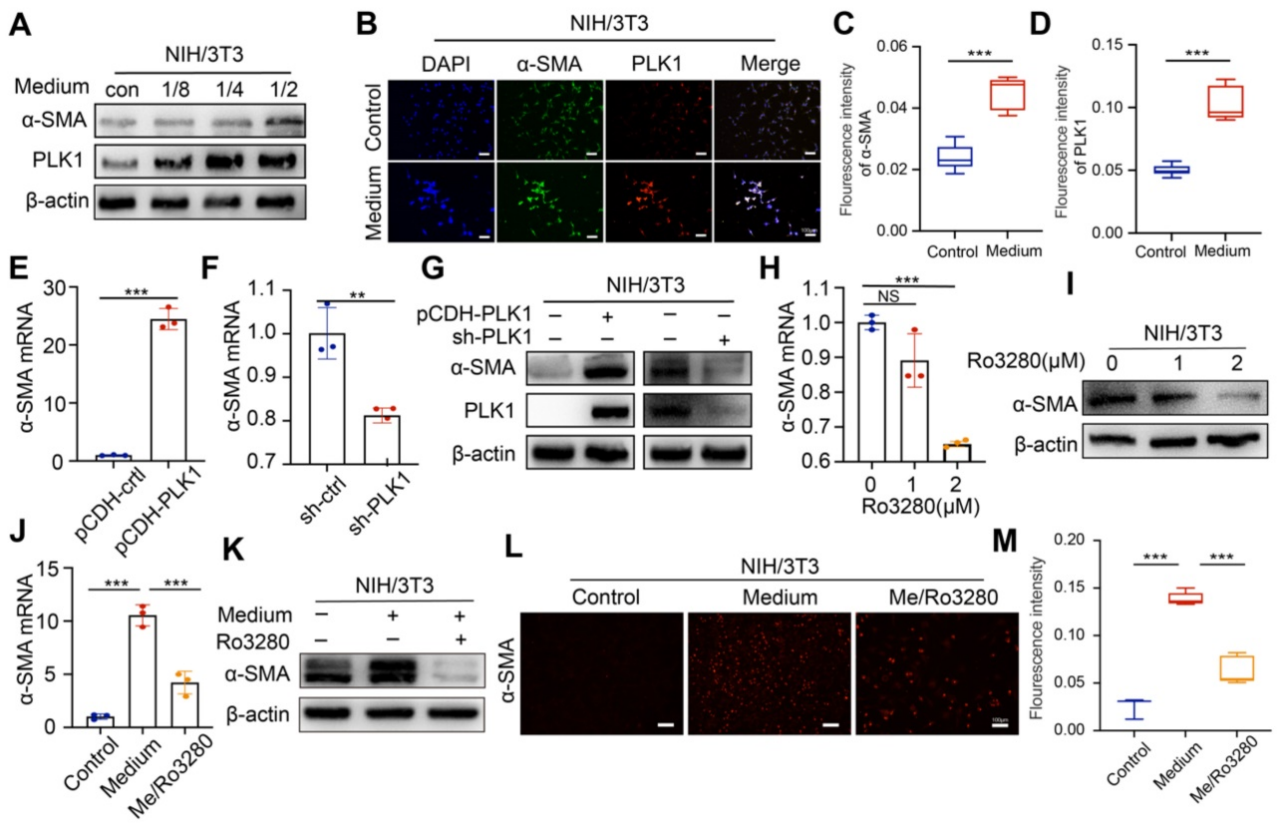
** Investigation of potential effect for PLK1 on CAFs.** A) The protein level evaluation of PLK1 and α-SMA in NIH/3T3 cells after different conditioned medium (collected from HuCCT1 cells) treatment by western blot. B) Expression level evaluation of PLK1 and α-SMA in activated NIH//3T3 cells by IF staining and quantitative results C-D). E-G) α-SMA mRNA and protein levels evaluation in PLK1 overexpression and deficiency NIH/3T3 cells by qRT-PCR and western blotting. H-I) α-SMA mRNA and protein levels evaluation after Ro3280 treatment in NIH/3T3 cells by qRTPCR and western blotting. J-K) α-SMA mRNA and protein levels evaluation after medium and Ro3280 treatment in NIH/3T3 cells. L) Expression level of α-SMA after medium and Ro3280 treatment in NIH/3T3 cells by IF staining and quantitative results M). scale bar: 100μm.

Conditioned medium from CCA tumor cells was collected and cocultured with mouse embryonic fibroblast (NIH/3T3) cells to obtain an activated phenotype (CAFs). Under the stimulation of conditioned medium, the expression of PLK1 showed a conditioned medium concentration- and incubation time-dependent increasing pattern (Figure 3A and Figure S8A), which also coincided with the expression trend of α-SMA. Immunofluorescence intensity for α-SMA and PLK1 increased after medium treatment, as shown in Figure 3B-D, further confirming the positive relationship between PLK1 and α-SMA. Next, we established stable PLK1-overexpressing and PLK1-knockdown NIH/3T3 cells to evaluate the correlation of PLK1 and α-SMA, as shown in Figure S8B-C. Notably, the α-SMA expression in PLK1-overexpressing NIH/3T3 cells increased significantly; in contrast, α-SMA expression was subsequently downregulated when PLK1 was knocked down (Figure 3E-G). As a matter of course, the inhibitory effect of Ro3280 on α-SMA expression in CAFs was then assessed by qRT-PCR and western blotting on activated NIH/3T3 cells, as shown in Figure 3H-I. Both the mRNA and protein levels of α-SMA decreased with increasing concentrations of Ro3280. Furthermore, medium collected from CCA cells and Ro3280 at a concentration of 2 μM was cocultured with NIH/3T3 cells. The mRNA and protein expression levels of α-SMA were evaluated by PCR, WB and immunofluorescence staining. As expected, NIH/3T3 only maintained the basal expression level of α-SMA. In contrast, the conditioned medium from CCA cells significantly activated the CAF phenotype, and Ro3280 reversed the activation state of CAFs (Figure 3J-M), indicating the potential to regulate stromal secretion in CCA tissue. Hepatic stellate cells LX2, the important cellular components in the process of liver fibrosis, showed the same increasing trend of α-SMA after conditioned medium stimulation, while the expression of α-SMA was decreased after Ro3280 treatment (Figure S9).

### Synthesis and characterization of HA-ODA and AA-HA-ODA

**Figure 4:**
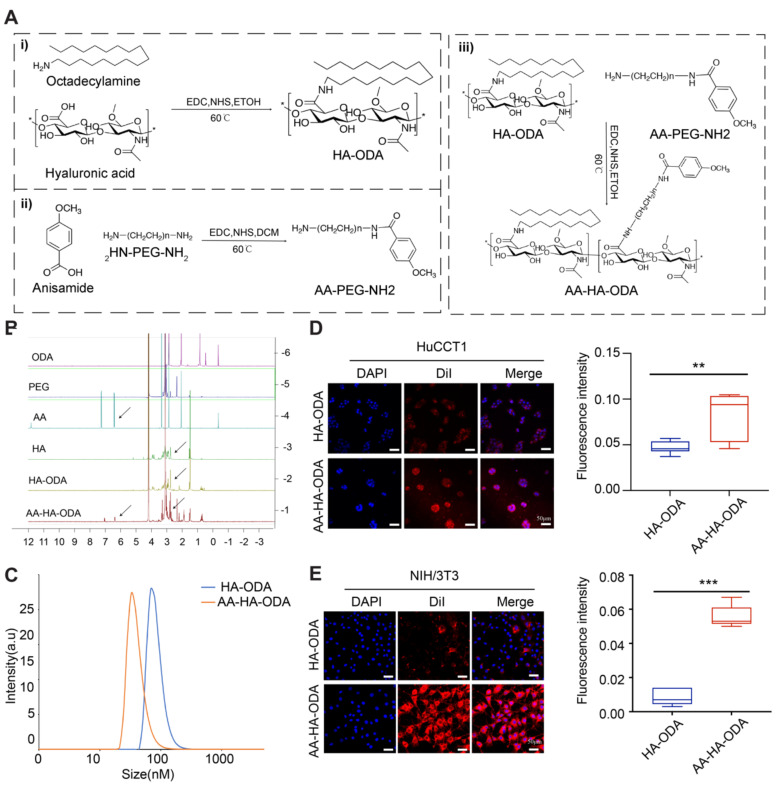
** The construction and characterization of AA-HA-ODA**. A) Flow chart of AA-HAODA construction. B) 1H NMR spectra of ODA, PEG, AA, HA, HA-ODA, AA-HA-ODA. C). Particle size distribution of HA-ODA and AA-HA-ODA. D-E). Confocal images of HA-ODA/DiI and AA-HA-ODA/DiI on HuCCT1 and NIH/3T3. The red fluorescence was the encapsulated DiI, cell nucleus was stained by DAPI (blue). Scale bar: 50μm. Quantification of the fluorescence intensity (right panel).

The therapeutic dilemmas in desmoplastic tumors caused by complicated crosstalk between tumor cells and stroma are often accompanied by a pathological stroma barrier, which reduces the accessibility of intratumorally delivered drugs. Studies have shown that anisamide derived ligands have high affinity for sigma receptors overexpressed in many malignant tumor cells and CAFs^46^. Based on sigma receptor-mediated endocytosis and EPR effect of tumor, nanodrugs modified with anisamide (AA) can promote drug accumulation in tumor mass through receptor-ligand interaction^28^. Western blot results showed that the expression of sigma receptor in cholangiocarcinoma tumor cells (HuCCT1 and RBE) and activated NIH/3T3 was significantly increased compared with normal intrahepatic bile duct epithelial cells HIBEpic (Figure S10). To evaluate the effectiveness of targeting mutual PLK1 for cholangiocarcinoma cells and CAFs *in vivo*, a targeted drug delivery system (AA-HA-ODA) with hyaluronic acid as the basic skeleton and PEG linked anisamide (AA) modified on the surface of micelles was constructed (Figure 4A). The chemical structure of AA-HA-ODA was analyzed by ^1^H-NMR spectrum as shown in Figure 4B. The peaks of AA-HA-ODA at about 7.0 ppm and 7.8 ppm were attributed to proton of AA, respectively. 2.8ppm was attributed to the -CH_2_- in PEG. Peaks at 1.0 ppm can be assigned to the proton of -CH_3_ and -CH_2_- from ODA. A serious peak at 4.4ppm and 3~4ppm could be attributed to the HA framework. The results jointly demonstrated the successful synthesis of AA-HA-ODA. According to the integrated peak area of specific proton of AA-HA-ODA from^ 1^H NMR spectrogram 47, 48, the substitution degrees of stearic amines in HA-DOA (the substituted ratio of sugar rings in HA) was calculated to be 10.6%, the conjugation ratio of AA-PEG-NH_2_ in AA-HA-DOA was recorded to be 14.2%.

The amphiphilic nature of polymeric micelles resulted in formation of nano-carrier in aqueous phase, with an apparent hydrodynamic diameter of approximately 40 nm for AA-HA-ODA respectively (Figure 4C). Surface positive nanoparticles will aggregate with negatively charged proteins in organisms, resulting in the inability of drug absorption. In order to further determine the *in vitro* stability of polymer micelles, HA-ODA and AA-HA-ODA were co incubated with 3% (w/v) fetal bovine serum, and the positively charged polymer polyethyleneimine (PEI) was used as the control group. As shown in Figure S11A, PEI was turbid after incubation with FBS, while HA-ODA and AA-HA-ODA were transparent. The turbidity at 350 nm of micelles were further investigated by UV-vis spectrophotometer, and the results showed that during the incubation process, the positively charged polymer PEI was turbid at an ultrafast rate and showed a time-dependent manner, while the turbidity of the micelles remained unchanged, showing great *in vitro* stability of AA-HA-ODA (Figure S11B). Dialysis method was performed to evaluate drug release of AA-HA-ODA/RO. Results in Figure S12 confirmed the reduced release profile of AA-HA-ODA/Ro. Then, the critical micelle concentration (CMC) of HA-ODA and AA-HA-ODA was recorded as 37.3μg/mL and 30.0 μg/mL by pyrene fluorescence method as shown in Figure S13.

### Validity of targeting and inhibiting PLK1 on cholangiocarcinoma cells and CAFs

Anisamide was reported to enhance cellular endocytosis through sigma receptor overexpressed on cell lines such as CAFs (activated NIH/3T3) and tumor cells 28. Fluorescence intensity of AA-HA-ODA on activated NIH/3T3 and HuCCT1 cells was 2.4- and 3.21- fold that of HA-ODA, as shown in Figure 4D-E, confirming the targeting capacity of AA-HA-ODA for CAFs and tumor cells, respectively. In order to better illustrate the role of anisamide and eliminate the interference of PEG in AA-HA-ODA, we also constructed another micelle PEG-HA-ODA. As shown in Figure S14, the fluorescence uptake of AA-HA-ODA is much higher in tumor cells and CAFs than that of HA-ODA and PEG-HA-ODA. Moreover, we conducted competitive uptake assay by preculture cells with free anisamide, which inhibited uptake of AA-HA-ODA /DII, possibly due to the occupation of sigma receptor, the binding site of anisamide on the cell surface (Figure S15). In summary, the uptake assay results together with competitive uptake assays indicated the association of the anisamide modification with enhanced internalization capacity of AA-HA-ODA in cholangiocarcinoma cells and activated NIH/3T3 cells.

To investigate the internalization capacity of AA-HA-ODA on normal cells in liver, hepatocytes cells (LO2) was adopted. As shown in Figure S16, compared with tumor cell HuCCT1 and activated NIH/3T3, the uptake intensity of AA-HA-ODA/DiI in hepatocyte LO2 is significantly lower. In addition, anisamide modification showed little uptake changes, jointly indicating that anisamide tend to increase the targeting capacity of sigma overexpressed HuCCT1 and activated NIH/3T3 cells.

### Penetration and antitumor efficacy of AA-HA-ODA/Ro on organoids and multicellular spheroids

**Figure 5:**
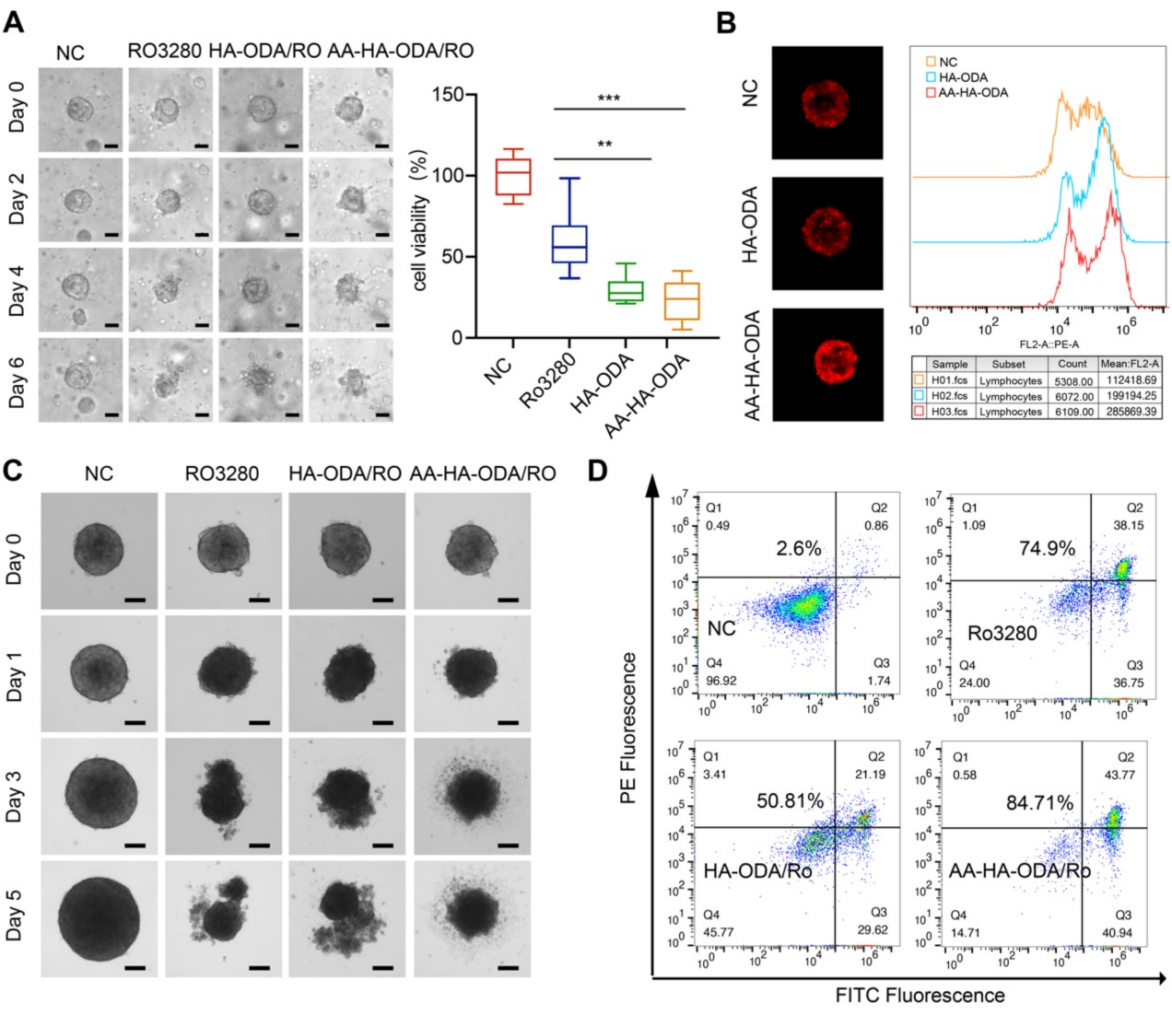
** Penetration and antitumor efficacy of AA-HA-ODA/Ro on multicellular spheroids.** A) Representative images of CCA organoids treated with Ro3280, HA-ODA/Ro and AA-HAODA/Ro for 1 weeks (scale bars: 50 μm, left panel) and quantification of cell viability from organoids (right panel). B) Penetration evaluation of AA-HA-ODA/Ro on MCTSs (NIH/3T3+HuCCT1) by confocal microscopy (left panel) and FACS (right panel). C) Representative images of MCTSs treated with Ro3280, HA-ODA/Ro and AA-HA-ODA/Ro, scale bars: 100μm. D) Antitumor efficacy of AA-HA-ODA/Ro were measured via flow cytometry by Annexin V/PI assay.

The antitumor efficacy of AA-HA-ODA/Ro *in vitro* was determined in CCA tumor cells and CAFs, as shown in Figure S17. Obvious cytotoxicity was observed in the two cell lines as a result of cell cycle arrest. Organoids are derived from primary tissues and have the capacity for long-term growth. Organoids contain varying levels of cellular complexity and physiologic similarities to native organ systems 49-51. Thus, an organoid model was first constructed to evaluate the efficacy of inhibiting PLK1, as shown in Figure 5A. After 6 days of Ro3280 treatment, the inhibition rate was less than 50%, while nearly 80% of the cells in the nanoform groups died, which verified the targeting ability of AA-HA-ODA/Ro. Meanwhile, HA-ODA/Ro also showed better antitumor effect compared with Ro3280, which could be attributed to the improved drug solubility and internalized Ro3280 by endocytosis 52. However, due to the integrality of extracellular matrix assembly and pathophysiological milieu conditions in tumor tissue 11, 53, 54, multicellular spheroids (MCTSs) composed of activated NIH/3T3 and tumor cells were constructed to evaluate the efficacy of the proposed targeting strategy by AA-HA-ODA/Ro. First, compared with the control group, the fluorescence intensity in AA-HA-ODA increased sharply as shown in Figure 5B. Notably, the fluorescence in HA-ODA group was weaker than that in AA-HA-ODA group. We have confirmed in our previous work that activated NIH/3T3 cells are mainly distributed outside the MCTSs 11, which simulats the pathological barrier and could hinder the ability of HA-ODA to reach internal tumor cells. The two peaks in the FACS results also indicated the heterogeneity of targeting and penetration, which jointly suggested the potential value for the strategy that sequentially targeting the stroma barrier and internal tumor cells for the delivery of PLK1 inhibitor *in vivo*.

When MCTSs were cultured for three days, PBS, Ro3280, HA-ODA/Ro and AA-HA-ODA/Ro (15 μM equivalent Ro3280) were added for further incubation, and images of the spheroids were captured by microscopy, as shown in Figure 5C. In general, Ro3280, HA-ODA/Ro and AA-HA-ODA/Ro significantly inhibited the growth of MCTSs after 1 day. With prolonged incubation time, necrosis in all drug treatment groups became much more pronounced. As shown in Figure 5D, the Annexin V/PI assay was further performed. A total of 74.9% of cells underwent apoptosis after Ro3280 incubation for 48 h. After AA-HA-ODA/Ro treatment, 84.71% of apoptotic cells were observed, verifying the superior antitumor effect of AA-HA-ODA/Ro. Notably, owing to the obstacle effect of the stroma barrier, the antitumor therapy of HA-ODA/Ro exhibited lower antitumor efficacy, which is consistent with the penetration result. An analogous tendency also appeared in MCTSs composed of RBE/A-NIH/3T3 cell lines, as shown in Figure S18, which emphasizes the effectiveness of the PLK1 inhibitor and the superior targeting capacity of AA-ODA-HA/Ro on CCA. Meanwhile, we verified the above results in hepatic stellate cell LX2. As shown in Figure S19, in the multicellular sphere penetration experiment composed of LX2 and HuCCT1, the fluorescence of AA-HA-ODA group was stronger than that of control group and HA-ODA group, and AA-HA-ODA/Ro significantly inhibited the growth of multicellular spheres and had stronger pharmacodynamic effect.

### Spatial distribution of dual targeting AA-HA-ODA *in vivo*

**Figure 6:**
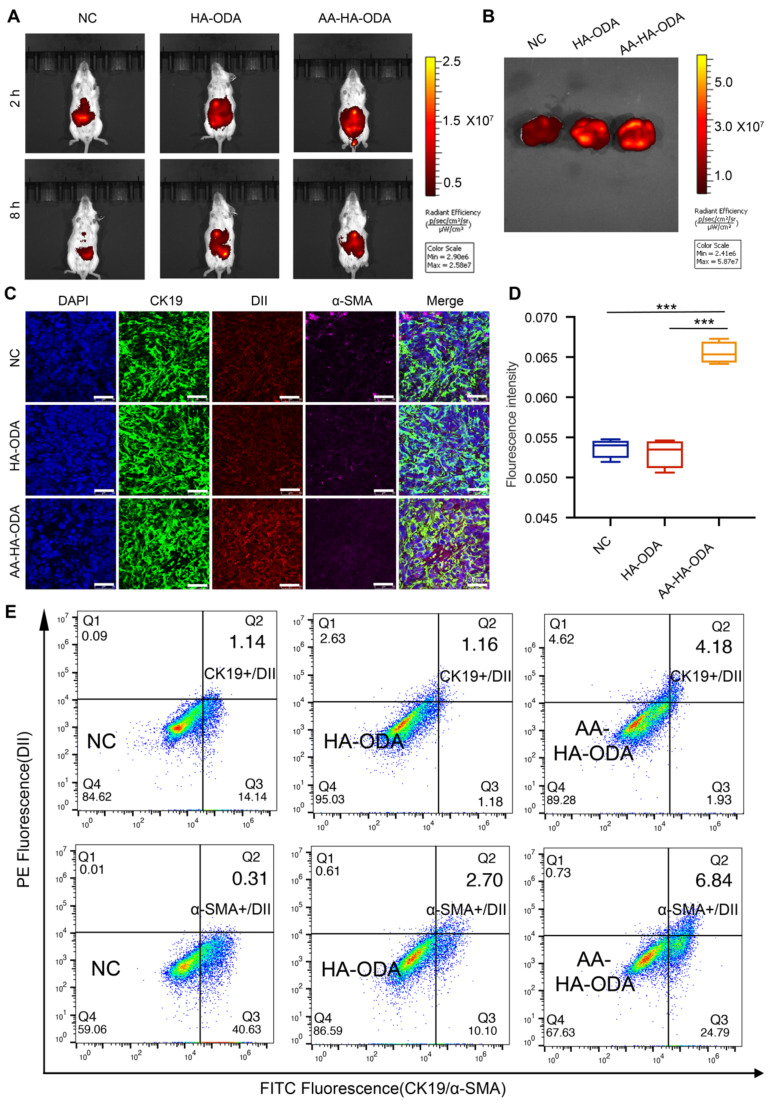
** Spatial distribution of dual targeting AA-HA-ODA *in vivo*.** A) *In vivo* distribution of ICG, HA-ODA/ICG and AA-HA-ODA/ICG by *In vivo* Imaging System. B) Amount of accumulated AA-HA-ODA in cholangiocarcinoma bearing liver. C) Distribution pattern of DiI, HAODA/DiI and AA-HA-ODA/DiI *in vivo*. α-SMA (pink) and CK19 (green) were marked by IF staining (24h). Dil was used as model drug. Scale bar : 50μm. D) Quantification analysis of the DiI positive area in liver. E) Flow cytometry evaluation of cells with both CK19 positive and AA-HAODA/DiI positive cells, α-SMA positive and AA-HA-ODA/DiI positive cells after 24h distribution, respectively.

The near-infrared fluorescent probe ICG (indocyanine green) was first used to label AA-HA-ODA to visualize the real-time distribution pattern in a primary murine cholangiocarcinoma model by an *in vivo* imaging system at predetermined time points (Figure 6A). Owing to the fact that the liver was the main metabolic organ, it was reasonable that strong fluorescence could be seen in the NC group. As expected, anisamide modification significantly promoted the specific distribution of tumors at both 2 h and 8 h. Afterward, the tumor-bearing liver was excised and visualized as shown in Figure 6B, which is consistent with the *in vivo* results. As the main metabolic organ, kidney has more fluorescence distribution than other organs, but it is weakly distributed in important organs such as heart, spleen and lung, which also reflects the great safety of micelles (Figure S20B).

To further explore the details of AA-HA-ODA *in vivo*, DiI encapsulated AA-HA-ODA was then applied to describe the profile of distribution. 24h after tail vein injection in the primary cholangiocarcinoma mouse model, strong fluorescence was observed in the liver tissue areas. Specifically, the tumor area (weaker fluorescence marked by a white dotted line) was surrounded by paracancerous tissue, and the fluorescence intensity of DiI was inversely correlated with the tumor location, demonstrating an urgency for intratumoral drug delivery (Figure S21). The solid components, such as cancer and stroma, collaboratively contributed to impeded drug delivery and diffusion 55, 56, and then CAFs and cholangiocarcinoma cells were stained for the spatialization of the delivered model drugs. As exhibited in Figure S22, rare green fluorescence labeled CK19 for cholangiocarcinoma cells overlapped with hollow-like weak red fluorescence for DiI, underlining the phenomenon of low permeability in intratumoral cholangiocarcinoma tissue. It is worth noting that a high density of CAFs was distributed around the margin of tumor tissue, which formed a grandly protective layer to hinder the distribution of drugs outside. In summary, the formed pathological stromal barrier might severely hinder the delivery of drugs *in vivo* and pose a huge challenge to confirm the effectiveness of Ro3280 *in vivo*.

Well-organized stromal networks can form “finger-like” projections, dividing tumor masses into distinct compartments to confine drugs to a limited space so that certain tumor regions are capable of regeneration 57. In fact, even with the aid of drug delivery system, very limited amounts of drugs can be delivered to target tissue as a result of various physiological/pathological barriers. Considering that the majority of drugs in blood circulation mainly accumulated in the liver, the signals of delivered DiI by nanoparticles in cholangiocarcinoma were very weak in comparison with those from the liver. Thus, red fluorescence was recaptured, which was confined to the CK19-positive area with a magnified view, as shown in Figure 6C. The strip-shaped DiI of the control group confirmed the heterogeneous distribution model so that recurrence normally became a clinical challenge. Owing to the stroma barrier around the tumor vessel, HA-ODA did not appear *in vivo* and decreased by 21.4% compared with the control group. Therefore, this phenomenon reminds us that the stromal barrier should be considered in the design of normal. Notably, the fluorescence intensity for AA-HA-ODA was 2.07 and 2.63 times that in the control and HA-ODA groups, respectively, as shown by the semiquantitative results in Figure 6D. Fluorescence intensity increased in AA-HA-ODA indicating the better penetration capacity after anisamide modification. The same tendency was also confirmed at 6h distribution group (Figure S23). In addition, tumor mass was excised and digested as single cells, CAFs were labeled with α-SMA, tumor cells were labeled with CK19, and determined by FACS. As shown in Figure 6E, a much higher proportion of α-SMA positive and AA-HA-ODA/DiI positive cells subset after 24h distribution confirmed the superior CAFs targeting capacity of AA-HA-ODA. The same results also appeared for AA-HA-ODA on cholangiocarcinoma cells.

With the continuous drug treatment, the stroma barrier was further investigated to further illustrate sequentially targeting capacity of AA-HA-ODA *in vivo*. Briefly, after HA-ODA/Ro administration, the fluorescence distribution pattern didn't exhibit obvious changes as shown in Figure S24. Conversely, the concentrated AA-HA-ODA/Ro ultimately reduced the CAFs positive area, resulting in tumor stroma collapsed so that the subsequent penetration of AA-HA-ODA/Ro for sigma receptor overexpressed cholangiocarcinoma cells can be deeper and more uniform. These results jointly confirmed that more AA-HA-ODA/Ro can be firstly delivered to CAFs as the results of the overexpressed sigma receptor on CAFs at pre-drug injection. Along with the elimination of stroma due to PLK1 inhibitor effect on CAFs after AA-HA-ODA/Ro injection, tumor cells inside tumor mass were exposed, deeper and more uniform permeation enforce the subsequently tumor cells targeting by AA-HA-ODA/Ro.

### Antitumor efficacy of AA-HA-ODA/Ro for inhibiting PLK1 on CCA *in vivo*

**Figure 7:**
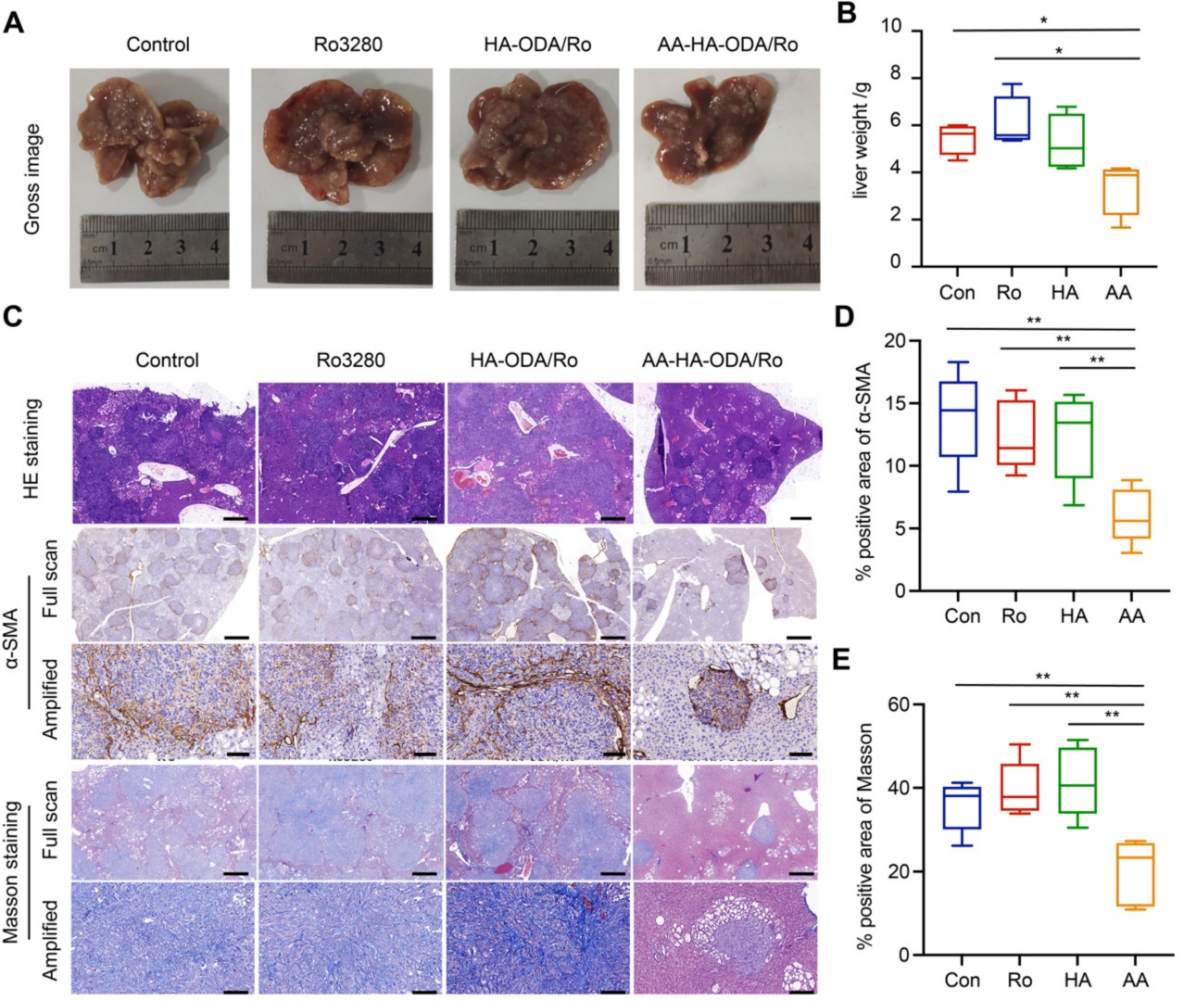
** Antitumor efficacy of Ro3280 in the virtue of AA-HA-ODA on CCA *in vivo*.** A-B) Images and weight of livers from CCA mouse models treated with Ro3280, HA-ODA/Ro and AAHA-ODA/Ro. C) Represntive images of liver by H&E, Masson staining and IHC for α-SMA. Scale bar for “Full sacn”: 50μm. Scale bar for Amplified”: 500μm. (D-E) Quantification of α-SMA and masson positive area in the gross image.

Mice were randomly divided into four groups: Control, Ro3280, HA-ODA/Ro and AA-HA-ODA/Ro. First, at the end of the experiment, the model mice were sacrificed, and the liver tissue was harvested and weighed to evaluate the antitumor efficacy, as shown in Figure 7A-B. The liver weight of the AA-HA-ODA/Ro group was significantly the lowest, indicating a better inhibitory effect on the proliferation of cholangiocarcinoma. However, single tumor cells targeting HA-ODA/Ro showed almost no significant change in liver weight compared with the control group, possibly due to the dense stromal barrier in the cholangiocarcinoma tissue. Subsequently, liver tissue was stained with HE, and the percentage of tumor-positive area in Figure 7C was consistent with the trend of liver weight.

Regarding the regulatory effect of Ro3280 on CAFs mentioned above, IHC and Masson staining were used to illustrate the characterization of α-SMA and collagen fibers in tumor tissues (Figure 7D-E). Abundant collagen and α-SMA in CAFs collectively profiled the outline of cholangiocarcinoma tissue, indicating that the cholangiocarcinoma tissue area has obvious desmoplastic characteristics. Notably, tumor cells were embedded into the tumor stroma (amplified sight), in which case administered Ro3280 might be confined to a limited space. Therefore, the stroma barrier made it difficult to verify the efficacy of PLK1 inhibitors *in vivo*. Unsurprisingly, compared with the control group, the therapeutic effect of Ro3280 was minimal. Consistent with the results from the antitumor assay, rich, compact and well-organized tumor stroma still existed for only tumor targeting HA-ODA/Ro as a result of the stroma barrier and extrema low penetration. A dramatic improvement in AA-HA-ODA/Ro was observed, as indicated by the absence of any outward evidence of pathological features, such as a contracting area of stroma and tumor nidus, demonstrating the effectiveness of PLK1 inhibiting strategies by a dual-targeting drug delivery system. As a marker of cell proliferation, ki67 was evaluated by immunohistochemistry. Ro3280 and HA-ODA/Ro had little effect on the proliferation of tumor cells, while AA-HA-ODA/Ro decreased the relative positive proportion of Ki67 (Figure S25), indicating the anti-proliferation effect of AA-HA-ODA/Ro. Tissue level apoptosis was further by tunel assay as shown in Figure S26. As indicated that the highest ratio of apoptosis positive occurred after AA-HA-ODA/Ro treatment in contrast with the Ro3280 and HA-ODA/Ro.

## Conclusion

With the deepening of the understanding of the microenvironment during CCA progression, the interaction between stroma and cholangiocarcinoma cells is considered to be a key factor in low efficacy and metastasis. Therefore, combination therapy for tumor cells and noncancerous cells has brought the light of treatment to patients. However, there are great challenges in the design of the dose and order of administration for the synergistic mechanism of different drugs. Therefore, this study proposes a strategy of sequentially inhibiting mutual targets in CAFs and cholangiocarcinoma cells and verifies its effectiveness. We first verified the pathological structure of CCA as rich and compact stroma by analyzing tissue samples from patients. Then, primary murine cholangiocarcinoma models were obtained by transfection of AKT/YapS127A with plasmids, and their pathologic characterization confirmed their suitability. Subsequently, biogenic analysis of human tissue and murine models together with *in vitro* and *in vivo* experimental data indicated that polo-like kinase 1 (PLK1) played a significant role in the cell cycle in cholangiocarcinoma cells. Furthermore, the results demonstrated that PLK1 also has a significant regulatory effect on the activity of CAFs. However, the therapeutic dilemmas of complicated crosstalk between tumor cells and stroma in desmoplastic tumors are often accompanied by a pathological stromal barrier, which reduces the accessibility of intratumorally delivered drugs. Therefore, dual-targeting AA-HA-ODA for CAFs and cholangiocarcinoma cells was constructed to deliver a PLK1 inhibitor sequentially to verify the effectiveness of inhibiting CAFs and PLK1 *in vivo*, which might provide enlightenment for multitarget treatment strategies and guidance for clinical cholangiocarcinoma treatment.

## Supplementary Material

Supplementary figures.

## Figures and Tables

**Scheme 1:**
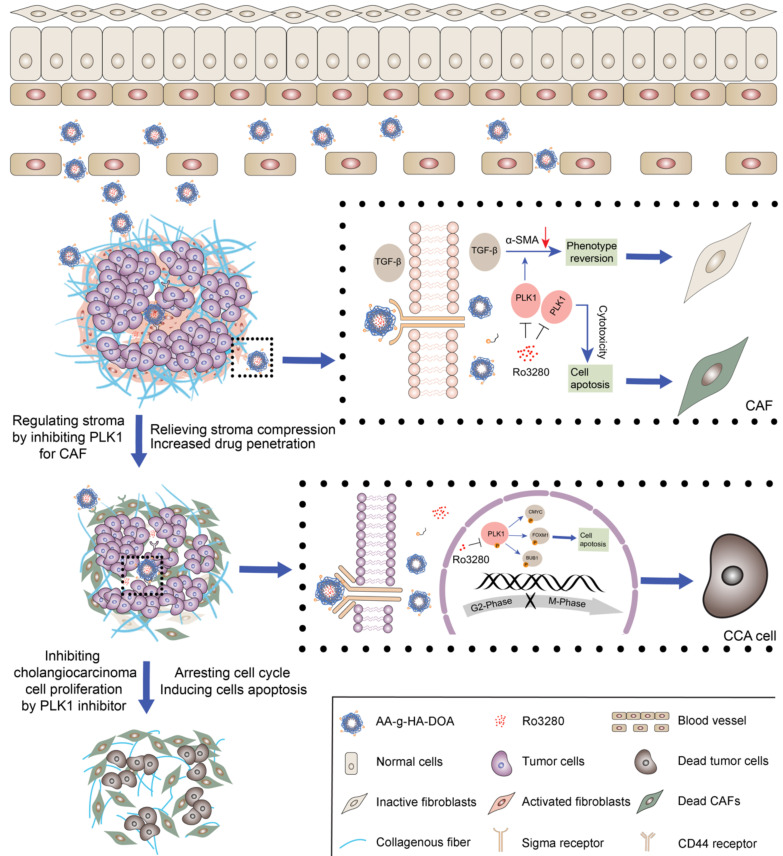
The graphic illustration of AA-HA-ODA/Ro regulating stroma barrier in desmoplastic CCA tumor masses and enhancing the synergistic antitumor treatment of CCA tumor cells.

## Abbreviations

CCA: cholangiocarcinoma
CAFs: cancer associated fibroblasts
PLK1: polo-like kinase 1
AA-HA-ODA: dual-targeting drug delivery system
HE: hematoxylin and eosin
DEGs: differentially expressed genes
MCTSs: multicellular spheroids
HA: Hyaluronic acid
NHS: 1-Hydroxy-5-pyrrolidinedione
EDC: 1-ethyl-3-(3-dimethylaminopropyl) carbodiimide
ICG: indocyanine green
DMSO: Dimethyl sulfoxide
PEG: Polyethylene glycol
DiI: 1,1'-dioctadecyl-3,3,3',3'- tetramethylindocarbocyanine perchlorate
