# Supplementary material for: Sequentially targeting and intervening mutual Polo-like Kinase 1 on CAFs and tumor cells by dual targeting nano-platform for cholangiocarcinoma treatment
Source: Theranostics. 2022 May 9;12(8):3911–27. doi: 10.7150/thno.70557 (PMC9131280; doi:10.7150/thno.70557)
Supplement: Supplementary file 1 — Supplementary figures. [file thnov12p3911s1.pdf]

Sequentially targeting and intervening mutual Polo-like Kinase 1 on CAFs and tumor cells by dual targeting nano-platform for cholangiocarcinoma treatment

Yue Zhou[1,2,*], Lei Xu[1,2,*], Zhangding Wang[1,2,*], Hongwen Liu[1,2], Xiang Zhang[1,2], Chuanjun Shu[4], Meng Zhang[1,2], Ting Wang[5], Xinyun Xu[5], Xiaohong Pu[5], Jian He[6], Pin Wang[1,2], Yudong Qiu[7], Guifang Xu[1,2,✉], Xiaoping Zou[1,2,✉], Yun Zhu[1,2,3,8,✉], Lei Wang[1,2,✉]

1. Department of Gastroenterology, Nanjing Drum Tower Hospital, The Affiliated Hospital of Nanjing University Medical School, Nanjing 210008, Jiangsu Province, China.

2. Department of Gastroenterology, Nanjing Drum Tower Hospital, Drum Tower Clinical Medical College of Nanjing Medical University, Nanjing 210008, Jiangsu Province, China.

3. Department of Pharmacy, Nanjing Drum Tower Hospital, Drum Tower Clinical Medical College of Nanjing Medical University, Nanjing 210008, Jiangsu Province, China.

4. Department of Bioinformatics, School of Biomedical Engineering and Informatics, Nanjing Medical University, Nanjing 211166, Jiangsu Province, China.

5. Department of Pathology, Nanjing Drum Tower Hospital, The Affiliated Hospital of Nanjing University Medical School, Nanjing 210008, Jiangsu Province, China.

6. Department of Nuclear Medicine, Nanjing Drum Tower Hospital, The Affiliated Hospital of Nanjing University Medical School, Nanjing 210008, Jiangsu Province, China.

7. Department of Hepatopancreatobiliary Surgery, Nanjing Drum Tower Hospital, The Affiliated Hospital of Nanjing University Medical School, Nanjing 210008, Jiangsu Province, China.

8. Nanjing Medical Center for Clinical Pharmacy, Nanjing 210008, Jiangsu Province, China.

✉ Corresponding authors: xuguifang@njglyy.com (Guifang Xu), zouxp@nju.edu.cn (Xiaoping Zou), zjuzhuyun@163.com (Yun Zhu), leiwang9631@nju.edu.cn (Lei Wang)

*These authors have contributed equally to this work.

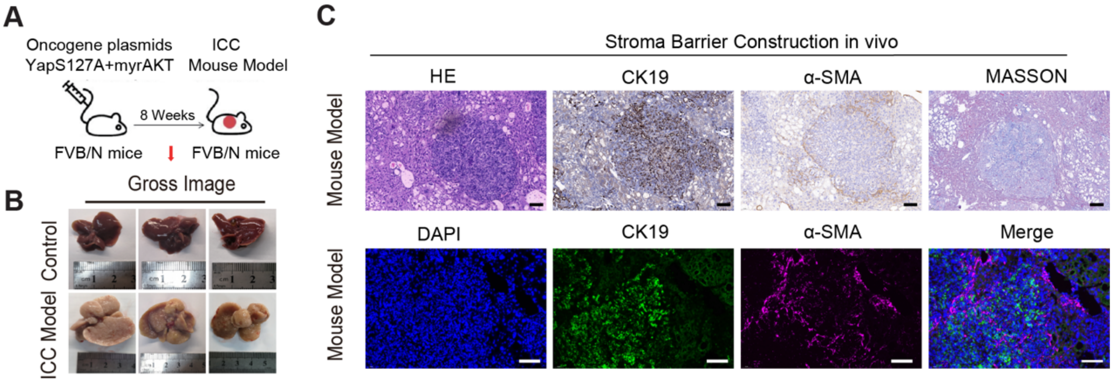

Figure S1. A) Schematic diagram of AKT/YapS127A induced CCA murine models construction. B) Images of cholangiocarcinoma on livers at 8 weeks post hydrodynamic injection respectively. C) HE, MASSON and IHC staining for CK19 and α-SMA (upper panel), Immunofluorescence analysis of CK19, α-SMA and DAPI (lower panel), Scale bar: 100μm

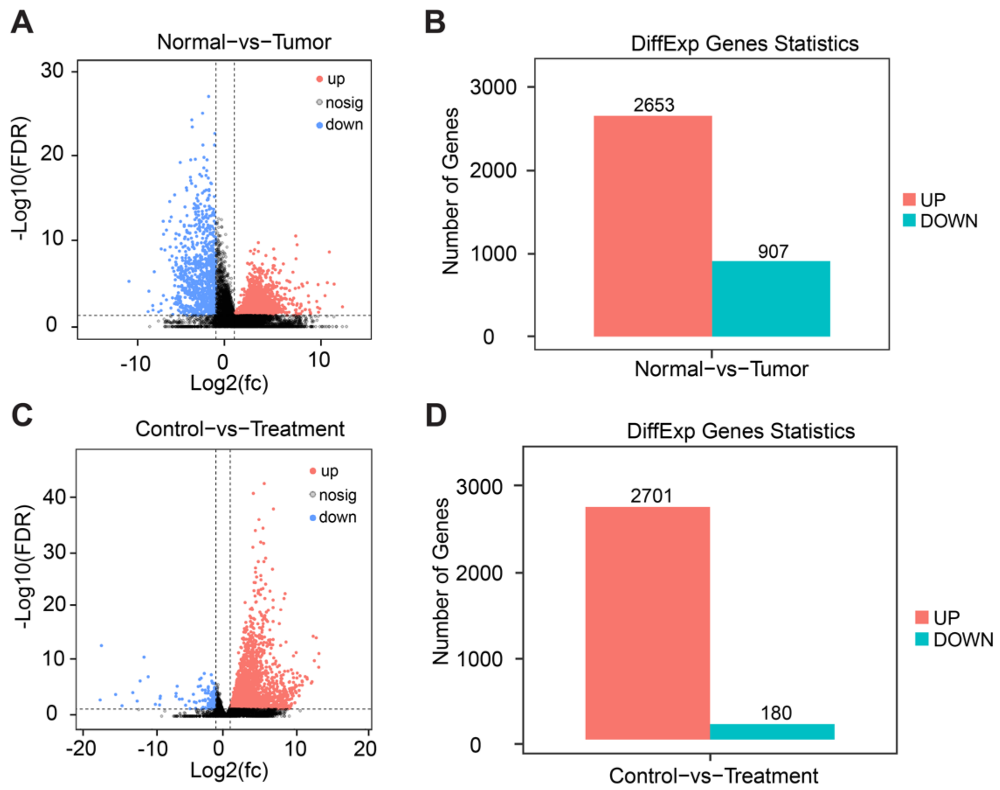

Figure S2. A) The volcano plots of differentially expressed genes (DEGs) for dataset Human patient tissues. B) Bar diagram of the number of DEGs. C) The volcano plots of DEGs for CCA mouse model. D) Bar diagram of the number of DEGs.

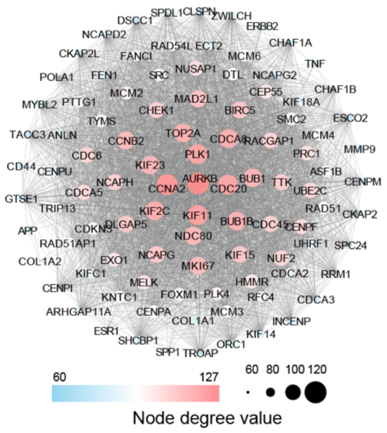

Figure S3. PPI network consisting of 100 nodes whose node degree ranked top 100 in the PPI network of DEGs. The network structure derives from Cytoscape following the application of the Organic layout.

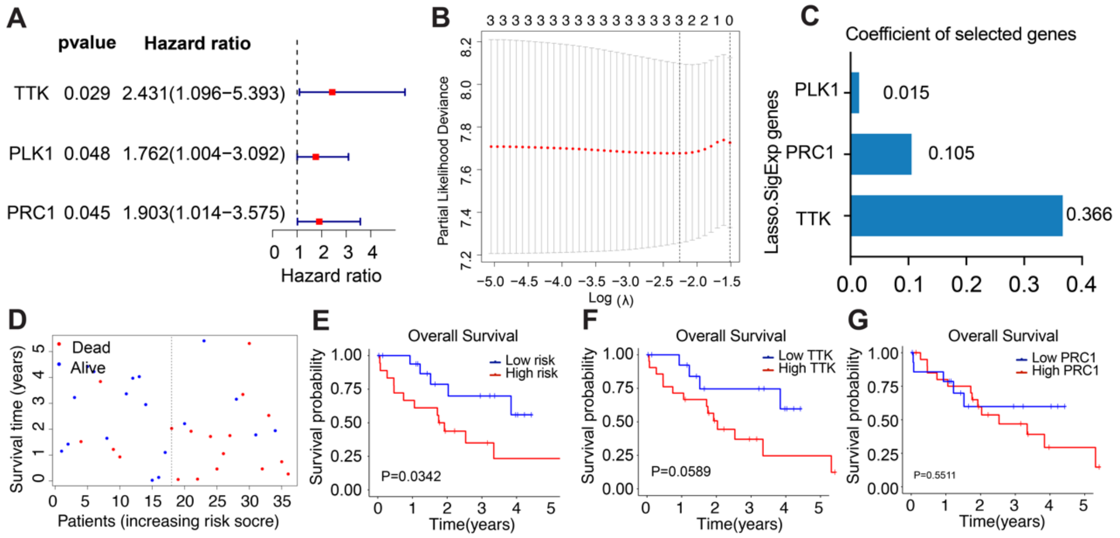

Figure S4. A) Forest plots showing the results of the univariate Cox regression analysis between cell cycle clustered gene expression and OS. B) Cross-validation for tuning parameters in the LASSO model, the vertical black line in the plot indicated the optimal log λ value. C) Selected genes and optimal weighting coefficients in the LASSO model. D) Distributions of survival time and risk score. E) Kaplan-Meier survival curve in the high- and low-risk groups. F) Kaplan-Meier survival curve in the high and low TTK expression groups. G) Kaplan-Meier survival curve in the high and low PRC1 expression groups.

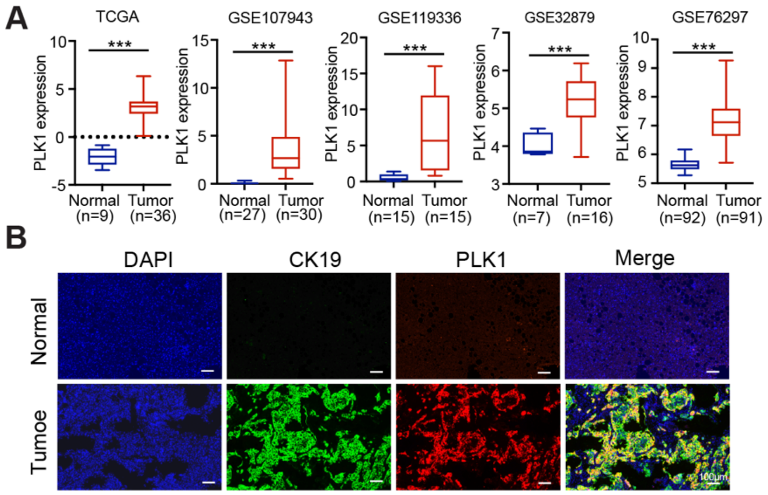

Figure S5. A) PLK1 mRNA expression in cancerous specimens compared noncancerous specimens base on TCGA data, GSE107943 dataset, GSE119336 dataset, GSE32879 dataset and GSE76297 dataset. B) Immunofluorescence analysis of CK19 (green), PLK1 (red) in CCA cancerous specimen compared with paired noncancerous specimen, cell nucleus was stained by DAPI (blue), scale bar: 100μm.

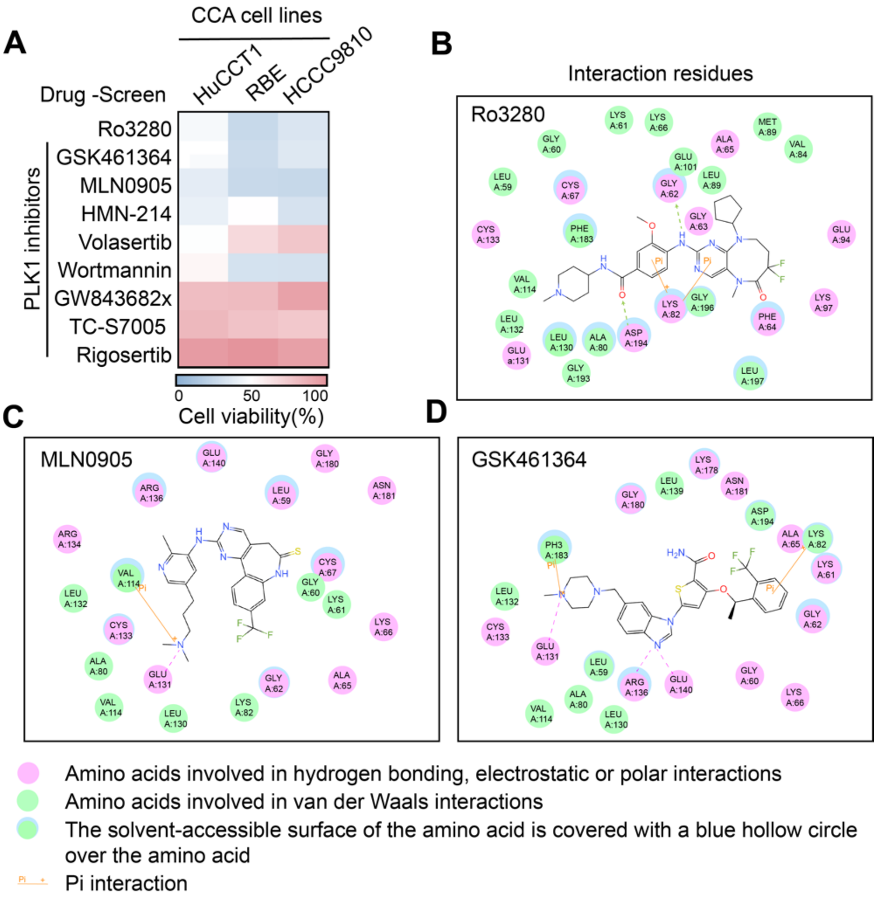

Figure S6. A) Evaluation of PLK1 inhibitors in all three CCA cell lines (HuCCT1, HCCC9810, and RBE). B-D) Interaction residues of Ro3280, MLN0905 and GSK461364 with PLK1 structure, respectively.

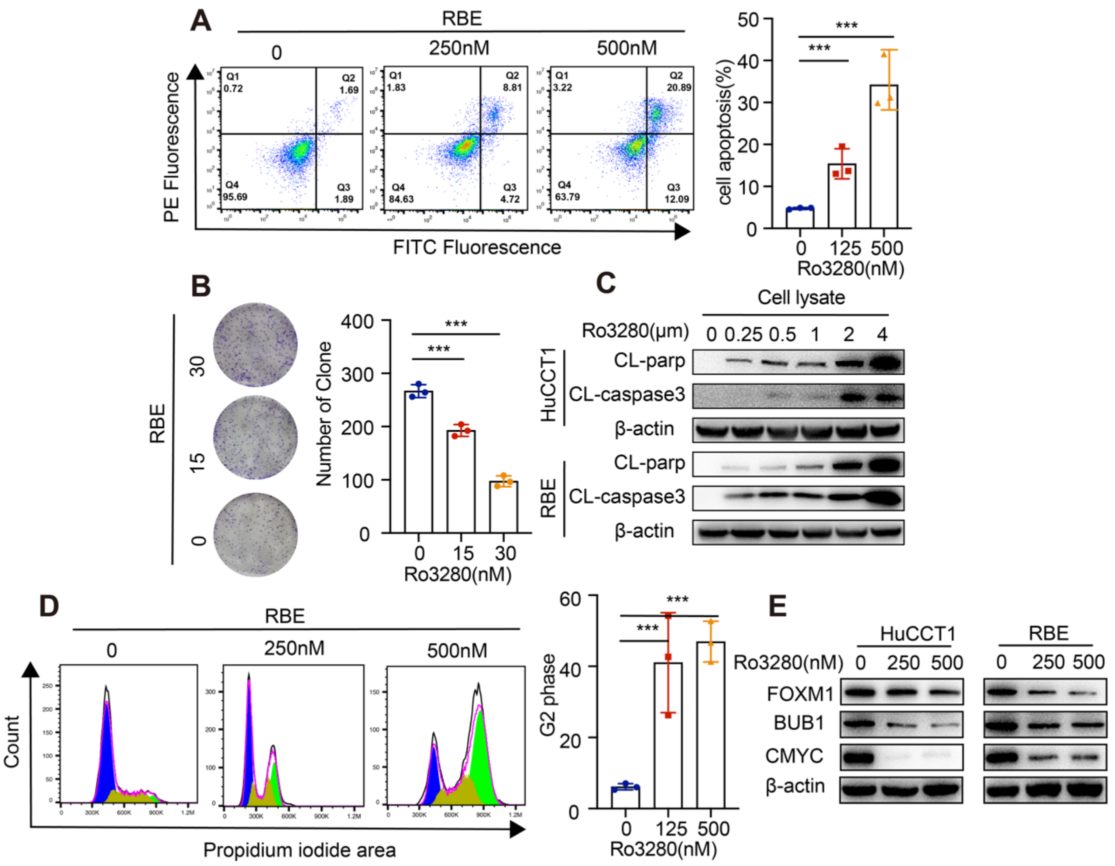

Figure S7. A) Cell apoptosis assays performed by flow cytometry after treatment of Ro3280 (left panel), quantification of the cell apoptosis assay results (right panel). B) Representative images of colony formation assay in RBE after treatment of Ro3280 (left panel), quantification of the colony formation assay results (right panel). C) The C-Caspase3 and C-Parp protein levels evaluation on HuCCT1 and RBE cells after Ro3280 treatment by western blotting. D) Cell cycle assays performed by flow cytometry after treatment of Ro3280 (left panel), quantification of the cell cycle assay results (right panel). E) FOXM1, BUB1, and CMYC protein expression were measured by western blot assay after treatment of Ro3280.

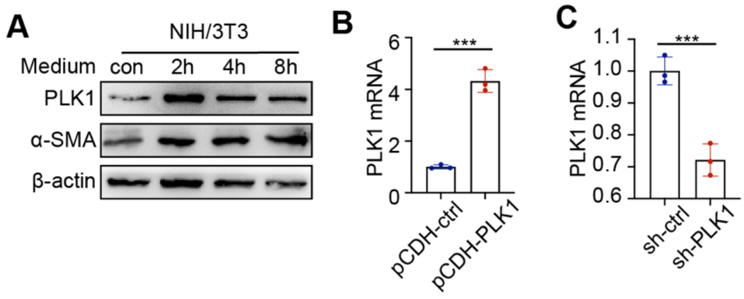

Figure S8. A) PLK1 and α-SMA protein expression were measured by western blot assay after different time conditioned medium. B-C) PLK1 mRNA evaluation in PLK1 overexpression and deficiency NIH/3T3 cells measured by qRT-PCR.

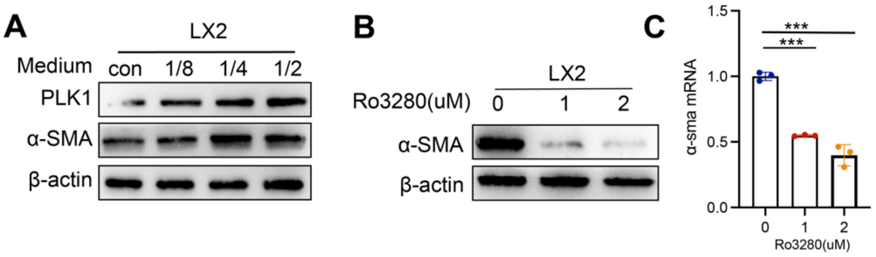

Figure S9. A) PLK1 and α-SMA protein expression were measured by western blot assay after different conditioned medium treatment. B-C) α-SMA mRNA and protein levels evaluation after Ro3280 treatment in LX2 cells measured by qRT-PCR and western blotting.

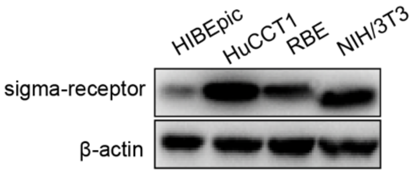

Figure S10. Sigma receptor protein expression were measured by western blot assay on different cell lines.

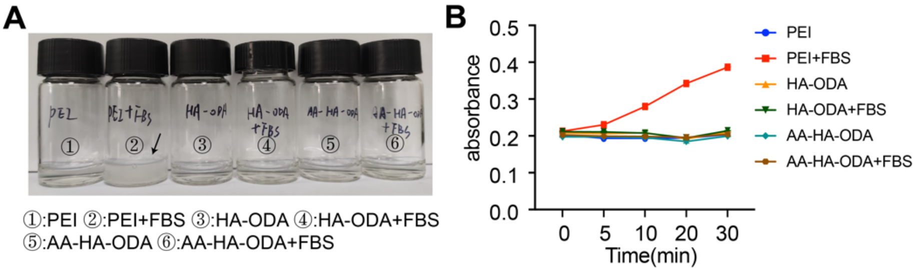

Figure S11. A) Images of PEI, HA-ODA and AA-HA-ODA incubated with 3% (w/v) FBS observed by camera. B) Absorbance of PEI, HA-ODA and AA-HA-ODA at 350nm after appointed incubation time with 3% FBS.

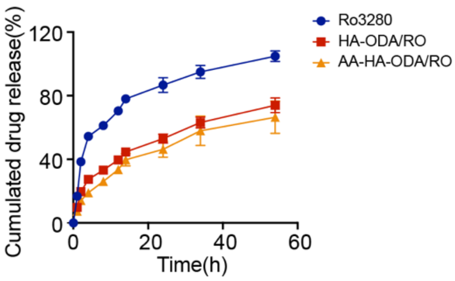

Figure S12. In vitro releasing profiles of Ro3280 free, HA-ODA/RO and AA-HA-ODA/RO.

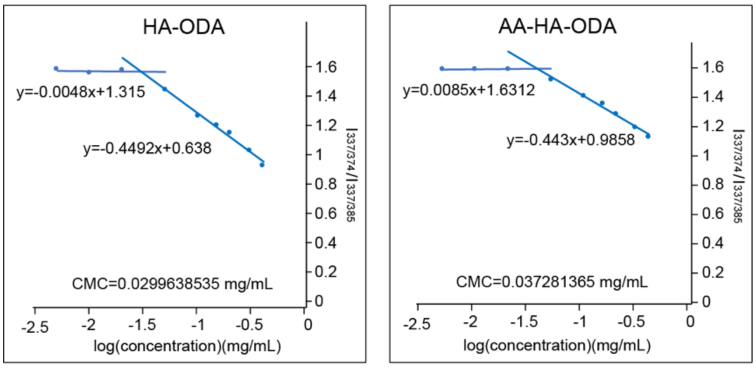

Figure S13. The I_1/I_3 ratio of pyrene in HA-ODA and AA-HA-ODA solution varies with LogC.

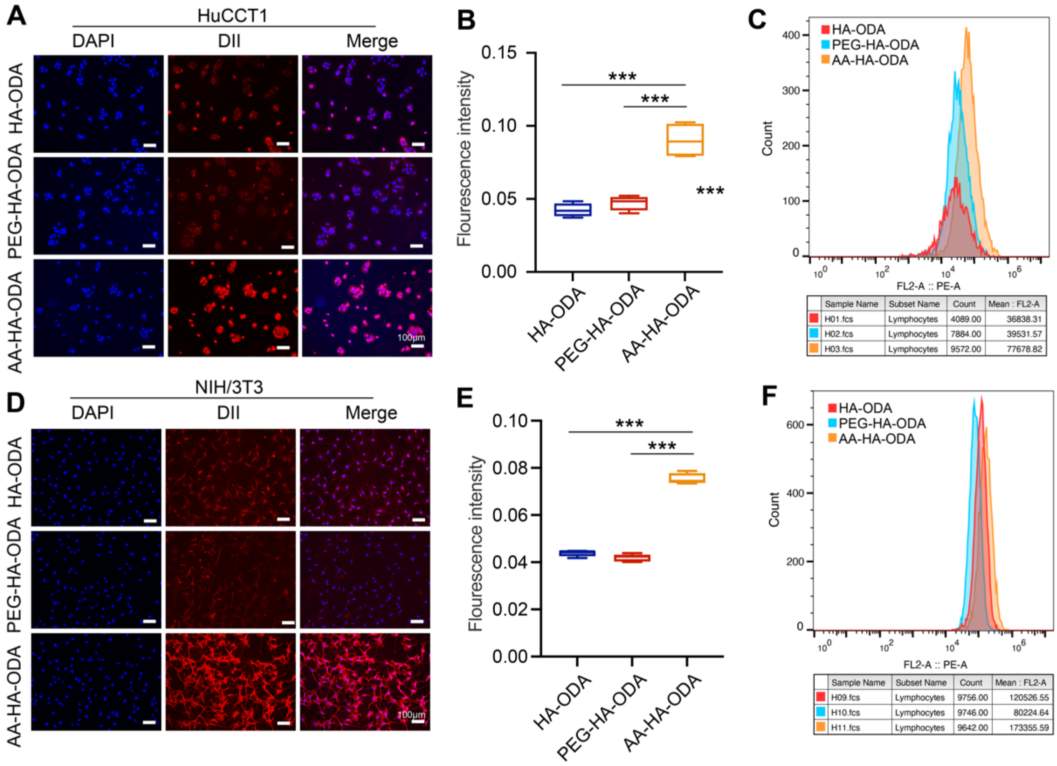

Figure S14. A) Confocal images of HA-ODA/DiI, PEG-HA-ODA/DiI and AA-HA-ODA/DiI on HuCCT1. B) Quantification of the fluorescence intensity. C) Fluorescence intensity performed by flow cytometry. D) Confocal images of HA-ODA/DiI, PEG-HA-ODA/DiI and AA-HA-ODA/DiI on NIH/3T3. E) Quantification of the fluorescence intensity. F) Fluorescence intensity performed by flow cytometry. scale bar: 100μm.

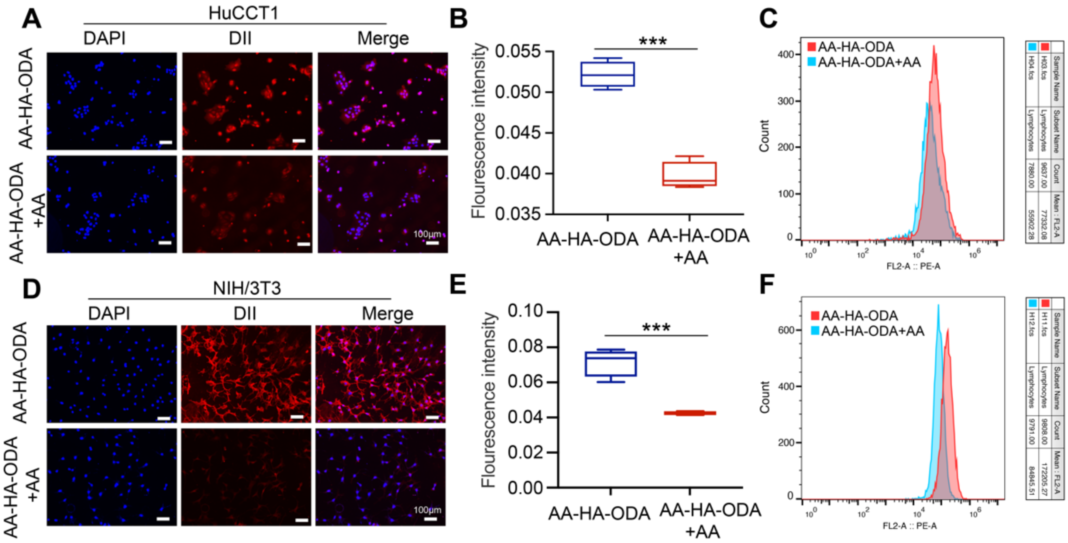

Figure S15. A) Confocal images of AA-HA-ODA/DiI on HuCCT1 after incubation with anisamide. B) Quantification of the fluorescence intensity. C) Fluorescence intensity performed by flow cytometry. D) Confocal images of AA-HA-ODA/DiI on NIH/3T3 after incubation with anisamide. E) Quantification of the fluorescence intensity. F) Fluorescence intensity performed by flow cytometry. scale bar: 100μm.

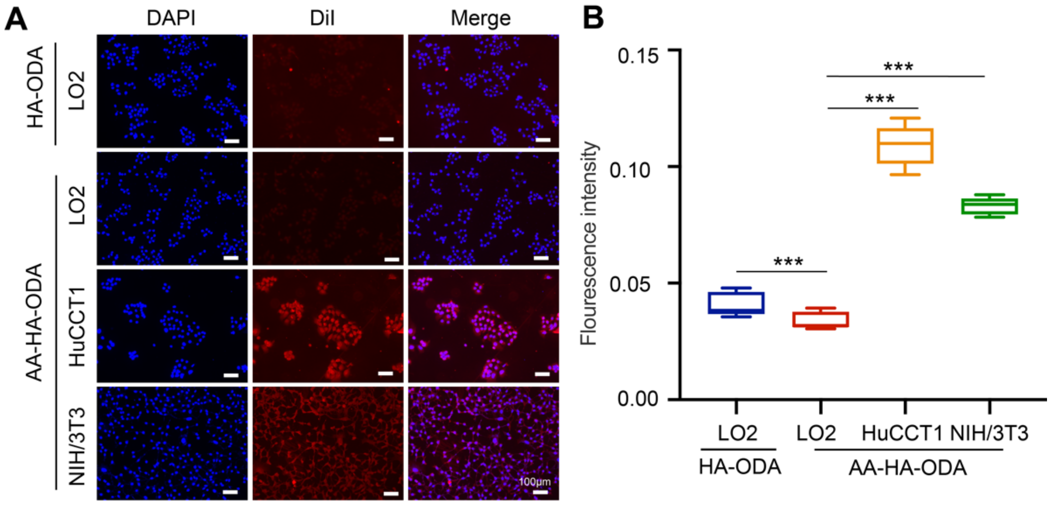

Figure S16. A) Confocal images of AA-HA-ODA/DiI on LO2, HuCCT1 and NIH/3T3. The red fluorescence was the encapsulated DiI, cell nucleus was stained by DAPI (blue). B) Quantification of the fluorescence intensity. scale bar: 100μm.

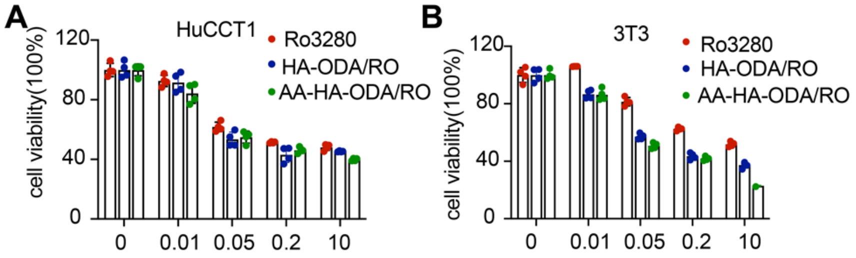

Figure S17. Relative cell viability of HuCCT1(A) and NIH/3T3(B) measured by CCK-8 assay after different drug treatments for 48h.

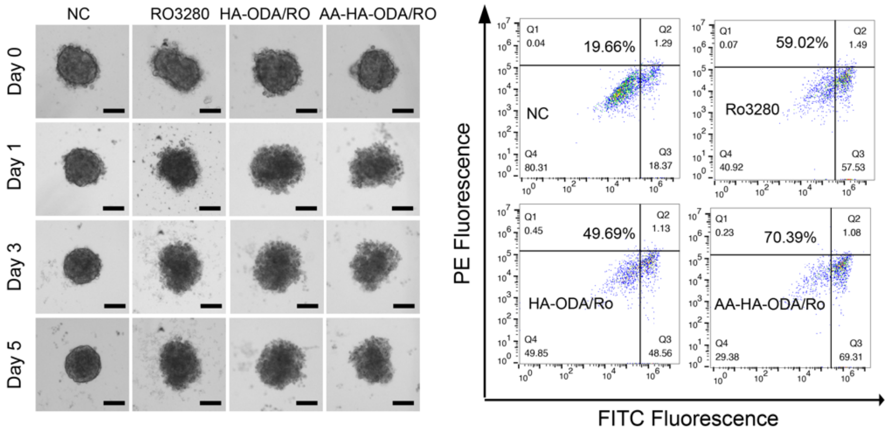

Figure S18. A) Representative images of MCTSs (NIH/3T3+RBE) after different drug treatments. B) Cell apoptosis assays of MCTSs performed by flow cytometry after different drug treatments. scale bar: 100μm.

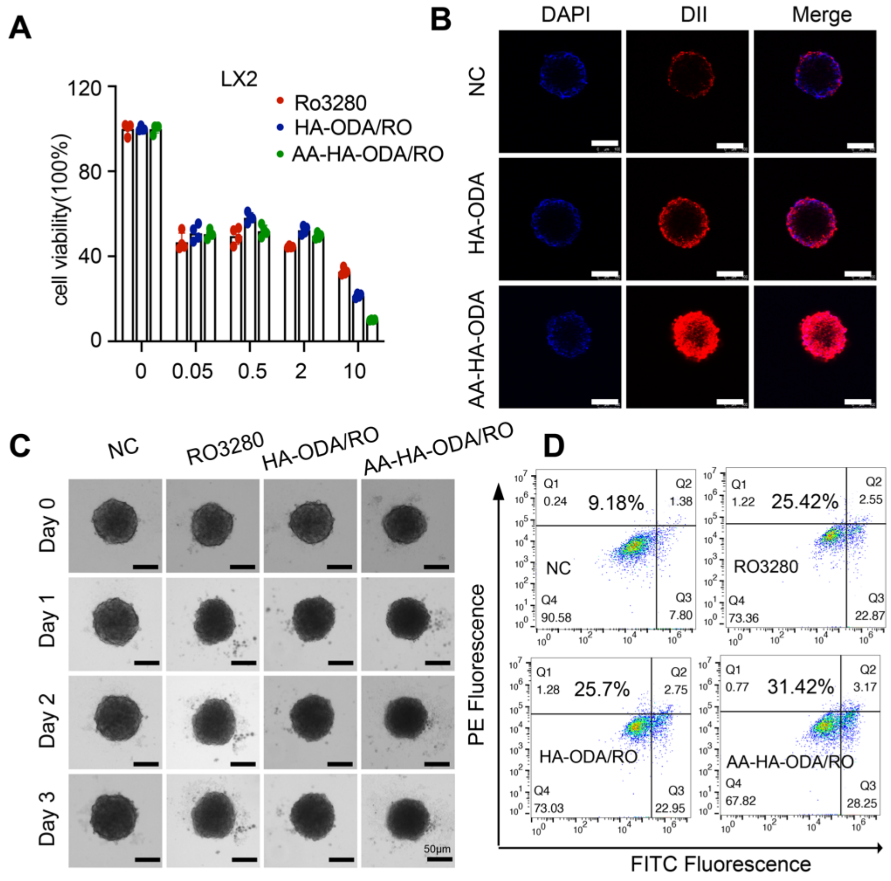

Figure S19. A) Relative cell viability of LX2 measured by CCK-8 assay after different drug treatments for 48h. B) Penetration evaluation of AA-HA-ODA/DII on MCTSs (LX2+HuCCT1) by confocal microscopy. scale bar: 50μm. C) Representative images of MCTSs treated with Ro3280, HA-ODA/Ro and AA-HA-ODA/Ro. D) Cell apoptosis assays of MCTSs performed by flow cytometry after different drug treatments. scale bar: 50μm.

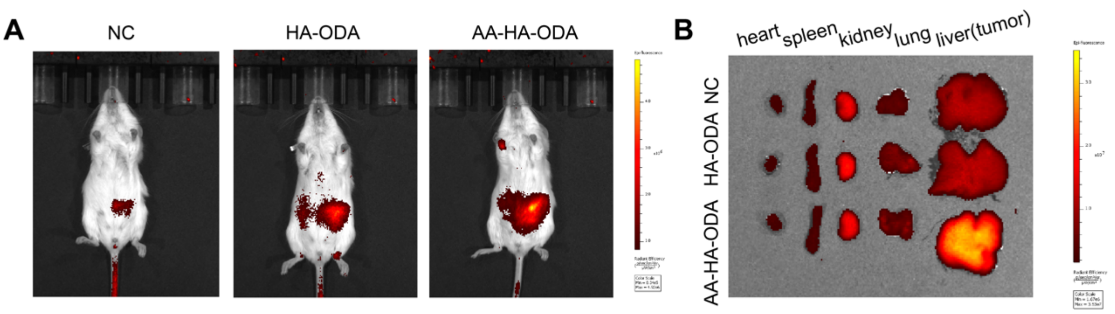

Figure 20. A) In vivo tracing delivered AA-HA-ODA by In vivo Imaging System. B) Amount of accumulated AA-HA-ODA in major organs.

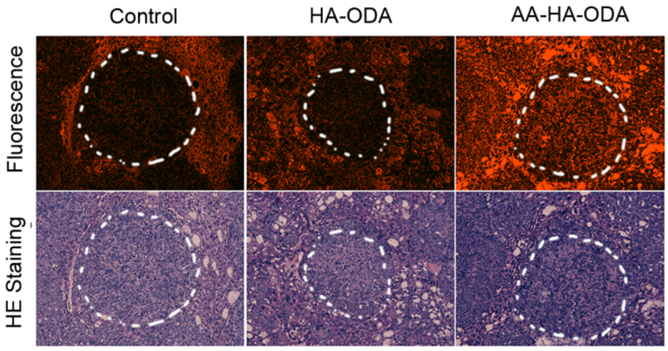

Figure S21. Fluorescence images of DiI loading AA-HA-ODA on murine CCA model.

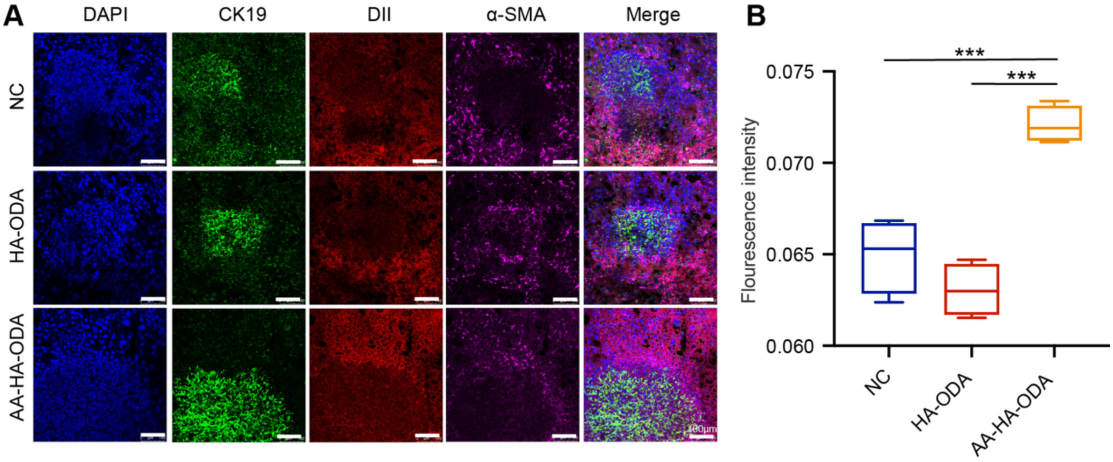

Figure S22. A) DiI was encapsulated to trace distribution pattern of AA-HA-ODA in vivo (24h). α-SMA (pink) and CK19 (green) were marked by IF staining. Dil was used as model drug. B) Quantification analysis of the DiI positive area in liver. scale bar: 100μm.

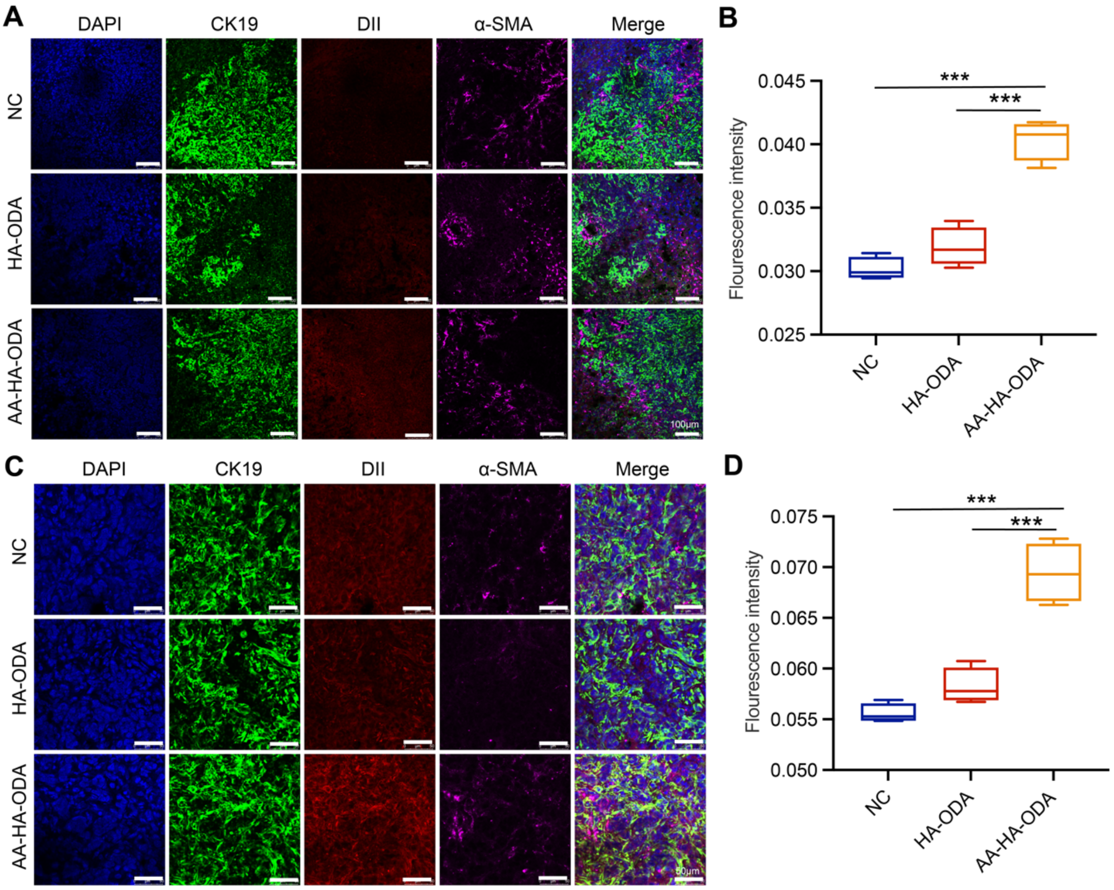

Figure S23. A) DiI was encapsulated to trace distribution pattern of AA-HA-ODA in vivo (6h). α-SMA (pink) and CK19 (green) were marked by IF staining. Dil was used as model drug. scale bar : 100μm. B) Quantification analysis of the DiI positive area in liver. scale bar: 100μm. C) DiI was en encapsulated to trace distribution pattern of AA-HA-ODA in vivo (6h). α-SMA (pink) and CK19 (green) were marked by IF staining. Dil was used as model drug. scale bar : 50μm. D) Quantification analysis of the DiI positive area in liver.

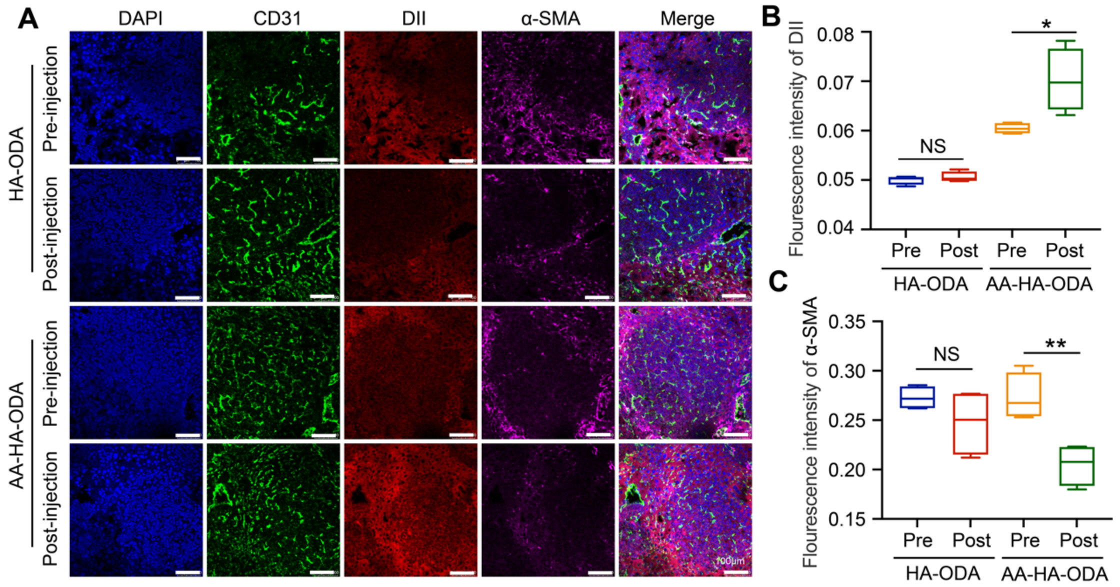

Figure S24. A) DiI was encapsulated to trace distribution pattern of AA-HA-ODA in vivo. α-SMA (pink) and CD31 (green) were marked by IF staining. Dil was used as model drug. B) Quantification analysis of the DiI positive area in liver. scale bar: 100µm.

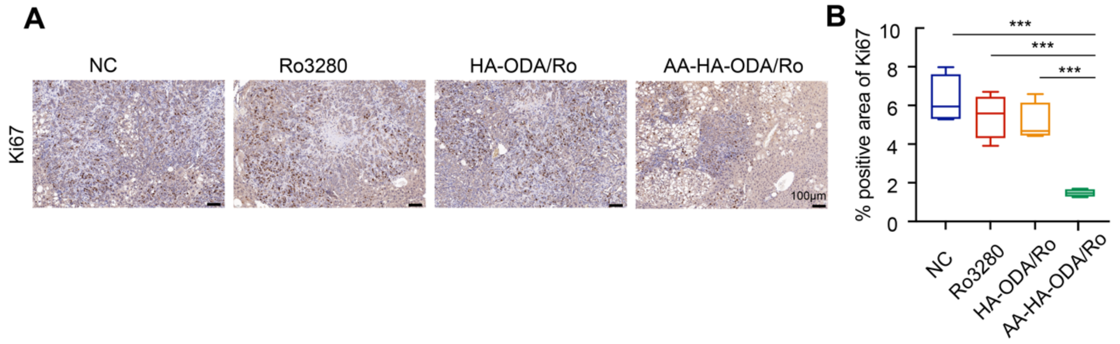

Figure S25. A) Represntive images of tumors by IHC staining for Ki67. B) Quantification of Ki67 positive area in the gross image. scale bar: 100µm.

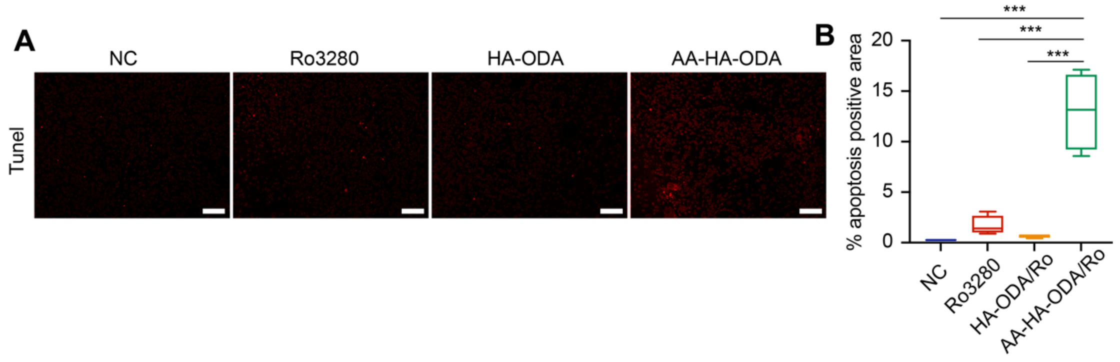

Figure S26. A) Tunel assay for the antitumor efficacy of AA-HA-ODA/Ro in vivo. scale bar: 100 µm. B) Semi-quantitative analysis of apoptosis positive area.
